# Parvovirus B19 NS1 protein induces cell cycle arrest at G2-phase by activating the ATR-CDC25C-CDK1 pathway

**DOI:** 10.1371/journal.ppat.1006266

**Published:** 2017-03-06

**Authors:** Peng Xu, Zhe Zhou, Min Xiong, Wei Zou, Xuefeng Deng, Safder S. Ganaie, Steve Kleiboeker, Jianxin Peng, Kaiyu Liu, Shengqi Wang, Shui Qing Ye, Jianming Qiu

**Affiliations:** 1 School of Life Sciences, Central China Normal University, Wuhan, China; 2 Department of Microbiology, Molecular Genetics and Immunology, University of Kansas Medical Center, Kansas City, Kansas, United States of America; 3 Department of Biotechnology, Beijing Institute of Radiation Medicine, Beijing, China; 4 Department of Pediatrics and Department of Biomedical and Health Informatics, The Children’s Mercy Hospital and University of Missouri Kansas City School of Medicine, Kansas City, Missouri, United States of America; 5 Department of Research and Development, Viracor-IBT Laboratories, Lee’s Summit, Missouri, United States of America; Cornell University, UNITED STATES

## Abstract

Human parvovirus B19 (B19V) infection of primary human erythroid progenitor cells (EPCs) arrests infected cells at both late S-phase and G2-phase, which contain 4N DNA. B19V infection induces a DNA damage response (DDR) that facilitates viral DNA replication but is dispensable for cell cycle arrest at G2-phase; however, a putative C-terminal transactivation domain (TAD2) within NS1 is responsible for G2-phase arrest. To fully understand the mechanism underlying B19V NS1-induced G2-phase arrest, we established two doxycycline-inducible B19V-permissive UT7/Epo-S1 cell lines that express NS1 or NS1^mTAD2^, and examined the function of the TAD2 domain during G2-phase arrest. The results confirm that the NS1 TAD2 domain plays a pivotal role in NS1-induced G2-phase arrest. Mechanistically, NS1 transactivated cellular gene expression through the TAD2 domain, which was itself responsible for ATR (ataxia-telangiectasia mutated and Rad3-related) activation. Activated ATR phosphorylated CDC25C at serine 216, which in turn inactivated the cyclin B/CDK1 complex without affecting nuclear import of the complex. Importantly, we found that the ATR-CHK1-CDC25C-CDK1 pathway was activated during B19V infection of EPCs, and that ATR activation played an important role in B19V infection-induced G2-phase arrest.

## Author summary

Human parvovirus B19 (B19V) is an autonomously replicating parvovirus with a marked tropism for human erythroid progenitor cells (EPCs) in the bone marrow and fetal liver. B19V infection results in several hematological disorders, which are a direct consequence of the infection and death of infected EPCs. One of the most significant sequelae of B19V infection of EPCs is G2-phase arrest, which has a marked effect on hematopoiesis. Here, we identify a key mechanism underlying B19V-induced G2-phase arrest. B19V NS1 is a global transactivator of cellular genes and as such activates ATR. ATR activation transduced signals to CDC25C and inactivated the cyclin B/CDK1 complex, which in turn prevented progression to cellular mitosis. However, NS1-induced ATR activation did not induce an obvious DNA damage response. Thus, B19V NS1 activates the ATR-CHK1-CDC25C-CDK1 pathway *per se* through its function as a transcriptional transactivator, highlighting the important role of ATR activation during G2-phase arrest of B19V-infected EPCs.

## Introduction

Human parvovirus B19 (B19V) is a small, non-enveloped, single stranded DNA (ssDNA) virus belonging to the genus *Erythroparvovirus* within the family *Parvoviridae* [1]. B19V causes fifth disease or slapped cheek syndrome in children; however, B19V infection can cause hematological disorders [2–6]. B19V infection of immunocompromised patients, such as those with HIV/AIDS, transplant recipients, and infants, leads to a persistent viremia that is associated with chronic anemia and pure red-cell aplasia. Acute B19V infection of patients experiencing increased destruction of erythrocytes, and therefore having a high demand for erythrocyte production (e.g., those with sickle-cell disease), can cause a transient aplastic crisis, whereas B19V infection of pregnant women can cause non-immune hydrops fetalis. B19V infects human erythroid progenitors at the burst-forming unit (BFU)- and colony-forming unit (CFU)-erythroid stages in the bone marrow [7–9] and fetal liver [10,11], which results in the death of infected cells [12–15]. Currently, there are no vaccines or specific antiviral therapeutics that can prevent or treat B19V-induced hematological disorders.

B19V has a linear single stranded DNA (ssDNA) genome of 5,596 nucleotides (nts), flanked on both sides by identical inverted terminal repeat sequences of 383 nts [16]. The internal coding region of the B19V genome contains a P6 promoter at the left hand of the genome. The left side of the genome encodes non-structural protein (NS1) and a 7.5-KDa protein, whereas the right side of the genome encodes two capsid proteins (VP1 and VP2), along with an 11-kDa protein (which uses a different open reading frame) [17–19].

The major B19V-encoded non-structural protein, NS1, is 671 amino acids long and has a molecular weight of about 78 kDa [17,20–22]. NS1 contains nuclear localization signals at amino acid residues 177–180 (KKPR) and 316–321 (KKCGKK); therefore, NS1 predominantly localizes in the nucleus of infected cells [23]. The N-terminus of NS1 contains a DNA-binding/nickase domain and the central region contains ATPase and a nucleoside triphosphate-binding motif, whereas the C-terminus contains putative transactivation domains [24,25]. NS1 is essential for replication of viral DNA [26], presumably due to its helicase and nickase activity [27]. NS1, with the help of transcription factors Sp1/Sp3, binds the P6 promoter of the virus to regulate viral gene expression [28,29]. In addition, NS1 transactivates several other host genes [30–32].

B19V infection inhibits differentiation of BFU- and CFU-erythroid progenitors, thereby arresting erythropoiesis [7,33]. It also induces apoptosis of infected erythroid progenitors [34]. Hydrops fetalis tissue infected with B19V exhibits characteristics of apoptosis [15], and fetal erythroid progenitors infected by B19V show ultrastructural features consistent with apoptosis [11]. B19V infection also induces cell cycle arrest at “G2-phase”, a point of the cell cycle at which cells contain 4N DNA [12,23,35,36]. “G2-phase” arrest of cells containing 4N DNA results in incorporation of BrdU (5-bromo-2’-deoxyuridine, a thymidine analog used as an indicator of DNA synthesis), which also suggests late S-phase arrest [37]. However, B19V NS1-induced G2 arrest of cells containing 4N DNA does not result in BrdU incorporation; hence, B19V NS1 induces “true” G2-phase arrest (BrdU^−^/DNA^4N^) [37]. B19V NS1 requires its predicted transactivation domain (TAD2; ^523^SSFFNLITP^531^), but not the endonuclease and helicase domains, to induce “G2-phase” arrest, which is independent of the p53 pathway [36]. B19V NS1 has also been reported to induce cell cycle arrest at G1-phase in NS1-expressing UT7/Epo-S1 cells [38]. Importantly, B19V infection induces a DNA damage response (DDR) that is essential for viral DNA replication [39], although the DDR has no effect on cell cycle arrest at “G2-phase” (BrdU^±^/DNA^4N^) [36]. Therefore, it is imperative to understand the mechanism underlying B19V NS1-induced “true” G2-phase arrest (BrdU^−^/DNA^4N^), in particular, the role of the NS1 TAD2 domain in the dysregulation of the cell cycle.

Here, we performed a genome-wide RNA-seq analysis of B19V-permissive cells (UT7/Epo-S1) to identify genes that are regulated by the NS1 TAD2 domain and function in NS1-induced G2-phase arrest. The results show that the TAD2 domain is predominantly responsible for the transactivation of cellular genes in NS1-expressing cells. Further pathway analysis revealed that NS1 TAD2 mediates activation of ATR (Ataxia-Telangiectasia Mutated and Rad3-related). We also found that activated ATR phosphorylates CDC25C (Cell Division Cycle 25C) at serine 216, which in turn inactivates the cyclin B1/Cyclin-Dependent Kinase 1(CDK1) complex and induces G2-phase arrest.

## Results

### The putative Transactivation Domain 2 (TAD2) is essential for NS1-induced G2-phase arrest

We previously showed that the B19V NS1 protein is a key factor for disrupting the cell cycle via the putative transactivation domain TAD2, and that viral DNA replication-induced DDR is not necessary for cell cycle arrest of cells containing 4N DNA [36]. B19V-infected cells that arrest at late S-phase and at G2-phase contain 4N DNA [37].

**Fig 1:**
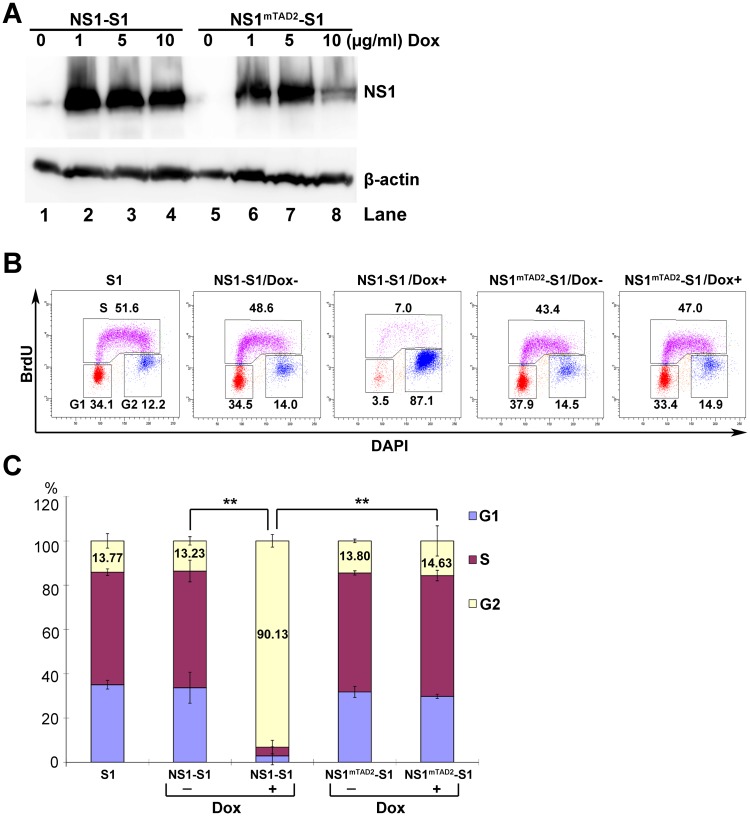
Putative Transactivation Domain 2 (TAD2) is essential for NS1-induced G2-phase cycle arrest. (A) Western blot analysis. NS1-S1 and NS1^mTAD2^-S1 cell lines were treated with different concentrations of Dox (1, 5, or 10 μg/ml) to induce expression of NS1 or NS1^mTAD2^, respectively. After 72 h, cell lysates were prepared and probed for NS1 and β-actin expression using anti-strep and anti-β-actin antibodies, respectively. (B) Cell cycle analysis. NS1-S1 and NS1^mTAD2^-S1 cells were treated with Dox (Dox+; 5 μg/ml) or not (Dox-) for 72 h and then incubated with BrdU, followed by treatment with 1N HCl. Treated cells were stained with an anti-BrdU antibody, and secondary antibody, and DAPI prior to flow cytometry. The numbers in each histogram are percentages of the cell populations with all BrdU-positive (S-phase), and BrdU-negative cell populations of 2N and 4N DNA content (G1- and G2-phase, respectively). (C) Statistical analysis. The percentage of cells at G1, S, and G2 are depicted in color. The percentages of the cells at G2-phase are shown in numbers, and compared in pairs as shown. **P<0.01.

To examine the mechanism underlying the function of the TAD2 domain during cell cycle arrest, we first established two doxycycline (Dox)-inducible UT7/Epo-S1 cell lines (NS1-S1 and NS1^mTAD2^-S1), which stably express B19V NS1 and mutant NS1^TAD2^, respectively, when exposed them to Dox. The optimal Dox concentration was 5 μg/ml, and incubation for 3 days led to expression of NS1 and NS1^mTAD2^ at similar levels in >95% of the cells (Fig 1A and S1 Fig). We next examined cell cycle changes in the two cell lines and found that nearly 90% of NS1-expressing cells arrested at G2-phase; by contrast, only 15% of the NS1^mTAD2^-expressing cells arrested at G2-phase, which is at the level similar to no induced cells (Dox-) or the UT7/Epo-S1 (S1) control (Fig 1B & 1C). This result clearly shows that TAD2 plays a role in NS1-induced cell cycle arrest at “true” G2-phase (BrdU^−^/DNA^4N^).

### The TAD2 domain is critical for NS1-mediated transactivation of cellular gene expression

**Fig 2:**
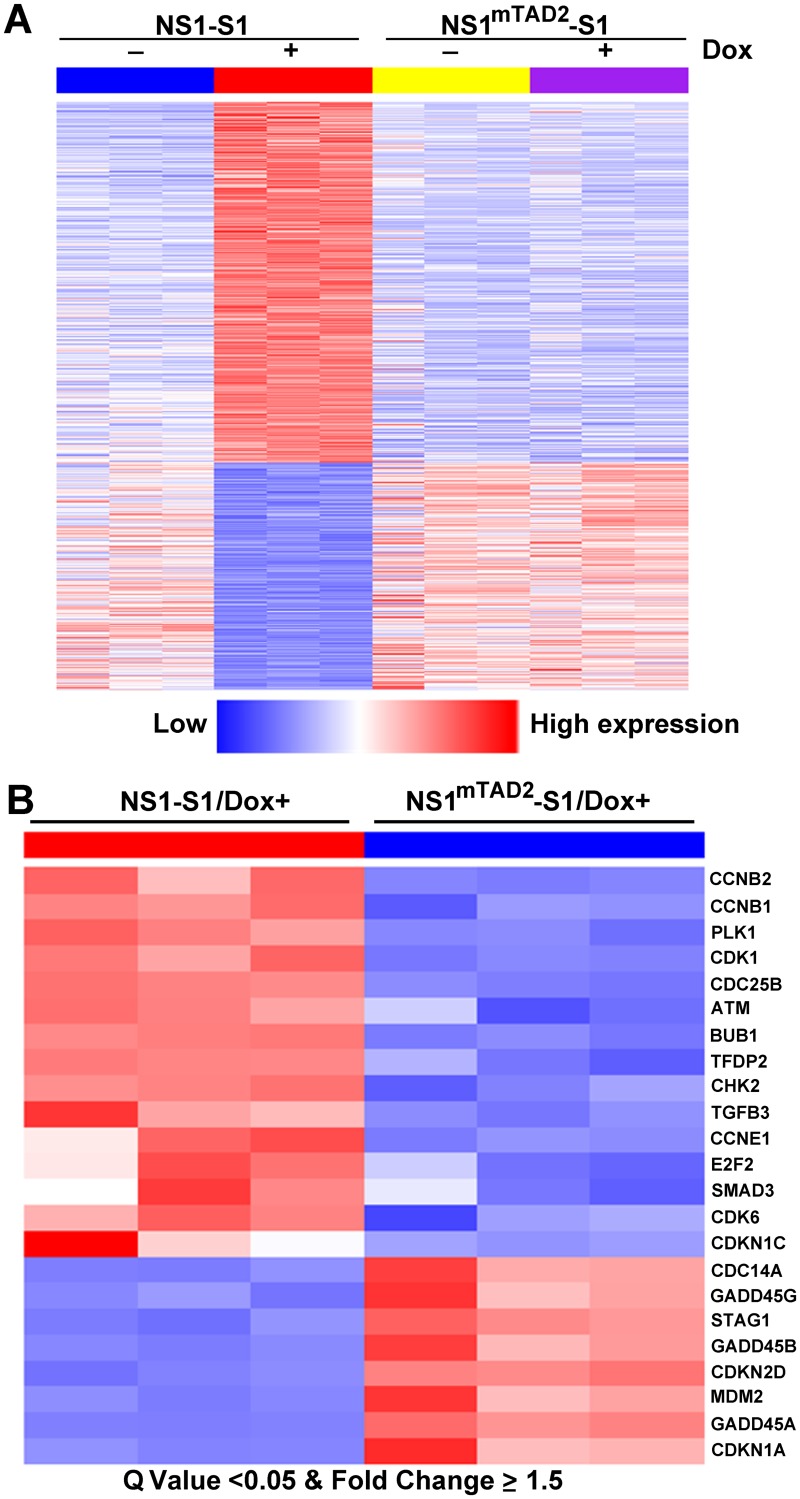
Transcriptomes of NS1-S1 and NS1^mTAD2^-S1 cell lines treated with or without Dox. (A) Heatmap of 1,770 genes differentially expressed between NS1-S1 and NS1^mTAD2^-S1 cell lines (Gene set 5). First, genes differentially expressed between NS1-S1 cell lines treated with and without Dox (NS1-S1 Dox+/-; Gene set 1) and between both NS1-S1 and NS1^mTAD2^-S1 cell lines treated with Dox (NS1-S1/Dox+ and NS1^mTAD2^-S1/Dox+; Gene set 2) were identified. Gene set 3 contains overlapped differential genes both in Gene sets 1 and 2. Next, genes differentially expressed between NS1^mTAD2^-S1 cells treated with and without Dox (NS1^mTAD2^-S1 Dox+/-) were identified as Gene set 4. Finally, Gene set 5, which represents the genes regulated by NS1 TAD2, was obtained by subtracting Gene set 4 from Gene set 3, and is shown. (B) Heatmap of 23 KEGG cell cycle genes showing significant differential expression between NS1-S1/Dox+ and NS1^mTAD2^-S1/Dox+ (Q value < 0.05 and fold change ≥ 1.5). Each column represents gene expression data for a NS1-S1 or a NS1^mTAD2^-S1 cell line (n = 3/cell line). Each row represents a gene. Red indicates increased expression. Blue indicates decreased expression.

The TAD2 domain was predicated using *in silico* analysis [36,40]. We then asked whether the TAD2 domain is a functional transactivation domain and whether it plays a role in the transactivation of cellular genes. To this end, NS1- and NS1^mTAD2^-expressing UT7/Epo-S1 cell lines were cultured in the presence/absence of Dox. Cells were collected, and total RNA was extracted and subjected to RNA-seq. The results revealed significant differential expression of 1,770 genes between NS1-S1 and NS1^mTAD2^-S1 cells, caused specifically by the NS1 TAD2 domain (Fig 2A). The heatmap showing the cellular transcriptome profile responding to NS1^mTAD2^ expression/no expression (NS1^mTAD2^-S1/Dox+ or Dox-) was similar to that showing non-NS1 expression (NS1-S1/Dox-); however, NS1-expressing cells (NS1-S1/Dox+) with differential expression of 1,770 genes showed upregulation of 1,064 genes and downregulation of 706 genes (Fig 2A). There were also 435 genes upregulated and 341 genes downregulated in mRNA levels of NS1^mTAD2^-S1 cells between presence and absence of Dox (Dox+/-), which are likely not due to the transactivation function of the NS1. Only 375 genes had significant changes between NS1-S1/Dox- and NS1^mTAD2^-S1/Dox- cell groups, which are likely due to the minor NS1 expression in these cells (Fig 1A, Dox 0). All RNA-seq data were deposited in Gene Expression Omnibus (accession no.: GSE83525).

**Fig 3:**
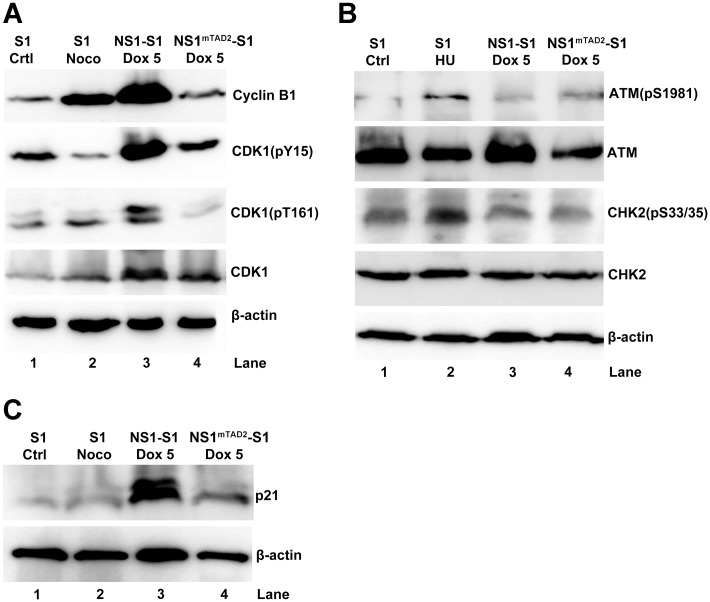
NS1 upregulates expression of cyclin B1, CDK1, and p21, but not expression of ATM and CHK2. NS1-S1 and NS1^mTAD2^-S1 cells were treated with Dox (5 μg/ml) and collected and lysed 72 h later. (A) Cell lysates were analyzed for expression of cyclin B1, phosphorylated CDK1 (CDK1(pY15) and CDK1(pT161), lower band [42]), and total CDK1 by Western blotting. (B) Cell lysates were analyzed for expression of ATM(pS1981), ATM, CHK2, CHK2(pS33/35), and β-actin by Western blotting. (C) Cell lysates were analyzed for expression of p21 by Western blotting with an anti-p21 antibody. β-actin was used as a loading control. Untreated UT7/Epo-S1 (S1) cells, S1 cells treated with nocodazole (Noco), and S1 cells treated with hydroxyurea (HU) were used as controls.

Further analysis of genes differentially expressed between NS1-S1 cells and NS1^mTAD2^-S1 cells in the presence of Dox using the KEGG program identified 23 KEGG cell cycle genes with fifteen genes that were upregulated by ≥ 1.5-fold and eight that were downregulated by ≥ 1.5-fold (Fig 2B). Among these, CCNB1/2 (cyclin B), CDK1, CDC25B, ATM (Ataxia Telangiectasia Mutated), CHK2 (Cycle Checkpoint Kinase 2), and CDKN1A (p21) play critical roles in regulating the cell cycle at G2-phase [41]. Therefore, we next performed Western blotting to examine expression of cyclin B, CDK1, ATM, CHK2, and p21. Expression of cyclin B1 or CDK1 was higher in NS1-expressing cells, as was CDK1 phosphorylation at both tyrosine (Y) 15 and threonine (T) 161 (CDK1(pY15) and CDK1(pT161), respectively), than in NS1^mTAD2^-expressing cells (Fig 3A, lanes 3 *vs*. 4, and S2A Fig), suggesting that CDK1 and cyclin B1 still form a complex. Noco-treated cells were used as a control for G2 arrest.

Of note, neither ATM nor CHK2 were significantly phosphorylated at serine (S) 1981 and S33/35, respectively, in NS1-S1 cells and NS1^mTAD2^-S1 cells, compared with positive control cells treated with HU (Fig 3B and S2C Fig), albeit ATM was expressed significantly high in NS1-S1 cells, compared with that from NS1^mTAD2^-S1 cells (S2B Fig). In agreement with previous reports, we found that p21, a cell cycle regulator that inhibits activity of the cyclin B1/CDK1 complex [43], was upregulated in NS1-expressing cells (Fig 3C) [32,38].

### The cyclin B1/CDK1 complex enters the nucleus, but exhibits reduced kinase activity, in NS1-expressing cells

**Fig 4:**
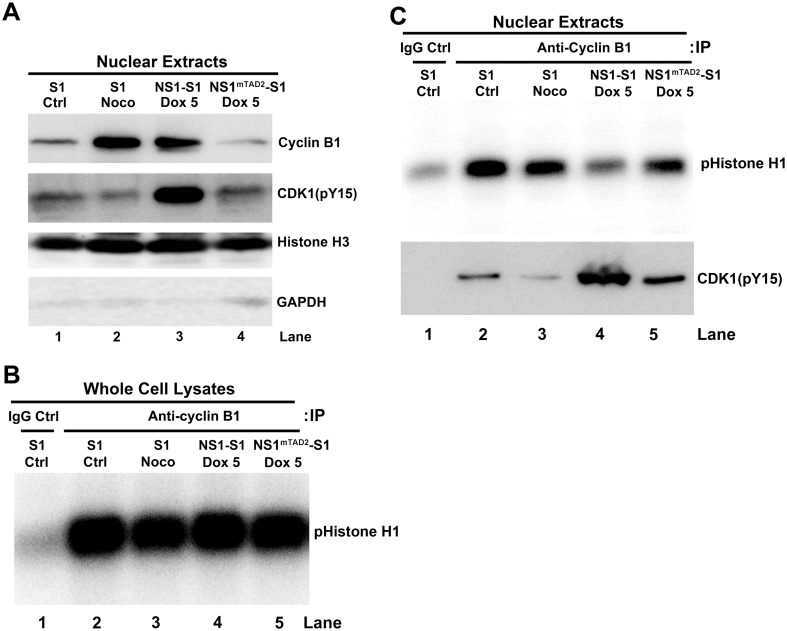
NS1 does not block the cyclin B1/CDK1 complex from entering the nucleus but it does inhibit its kinase activity. (A) Western blot analysis. NS1-S1 and NS1^mTAD2^-S1 cells were treated with Dox for 72 h, collected, and lysed, and the nuclei were extracted. Western blot analysis was then performed to detect cyclin B1 and phosphorylated CDK1(pY15), as well as nuclear histone H3 and cytoplasmic GAPDH (Glyceraldehyde-3-Phosphate Dehydrogenase). Nuclear extracts from S1 cells and S1 cells treated with nocodazole (Noco) were loaded as controls. (B&C) *In vitro* CDK1 kinase assay. Equivalent amounts of proteins derived from whole cell lysate (B) or nuclear extracts (C) from S1 cells, S1 cells treated with Noco, and Dox-induced NS1-S1 and NS1^mTAD2^-S1 cells were immunoprecipitated with anti-cyclin B1-crosslinked protein A/G Plus agarose beads for *in vitro* CDK1 kinase assay. The final products were resolved on a 12% SDS-polyacrylamide gel. The gel was then dried prior to autoradiography, and the phosphorylated histone H1 (pHistone H1) is indicated. (C) Lower panel: Co-Immunoprecipitation (Co-IP) of cyclin B1 and CDK1(pY15) in the nuclear extracts. Nuclear extracts prepared from S1, S1/Noco, NS1-S1/Dox+, and NS1^mTAD2^-S1/Dox+ cells were immunoprecipitated with an anti-cyclin B1 antibody. The eluted proteins were analyzed for CDK1(pY15) by Western blotting. Normal mouse IgG (IgG Ctrl) was used as negative control of immunoprecipitation of control S1 cell extracts.

We next focused on the cyclin B1/CDK1 complex, the key mitotic regulator of the G2/M transition phase [44,45]. For a cell to enter the mitotic phase, the cyclin B1/CDK1 complex must be imported into the nucleus [46,47]. It has been reported that B19V NS1 prevents the import of the cyclin B1/CDK1 complex to the nucleus [35]; therefore, we first asked whether NS1-induced G2 arrest allows the complex to enter the nucleus. Both cyclin B1 and CDK1(pY15) that is the inactive form of CDK1 [41] were detected in nuclear extracts of NS1-expressing cells (Fig 4A), clearly showing that NS1 does not prevent nuclear import of the cyclin B1/CDK1 complex, and there were much less CDK1(pY15) in the nuclear extracts of NS1^mTAD2^-S1 cells. This result suggests that the nuclear transport of cyclin B/CDK1(pY15) is likely TAD2 domain-dependent. Next, we used an *in vitro* kinase assay to examine cyclin B1/CDK1 complex activity in the nucleus. We found that whole cell lysates derived from each cell line showed similar kinase activity in the phosphorylation of histone H1 (Fig 4B). However, the nuclear extract prepared from NS1-expressing cells in G2-phase arrest showed reduced CDK1 kinase activity (Fig 4C, lane 4, pHistone H1), which is due to the high level of CDK1(Y15) immunoprecipitated from the nuclear extracts of NS1-S1 cells (Fig 4C, lane 4, CDK1(pY15)).

Taken together, these results show that NS1 induces the expression of both cyclin B1 and CDK1, and, also, the cyclin B1/CDK1 complex enters the nucleus; however, the complex is presented in an inactive form that has CDK1(pY15), which prevents the transition from G2- to M-phase.

### NS1 TAD2 domain activates ATR-CHK1, p38-MK2, and MARK3 kinases that potentially regulate CDC25C activity but without induction of hallmarks of DDR

**Fig 5:**
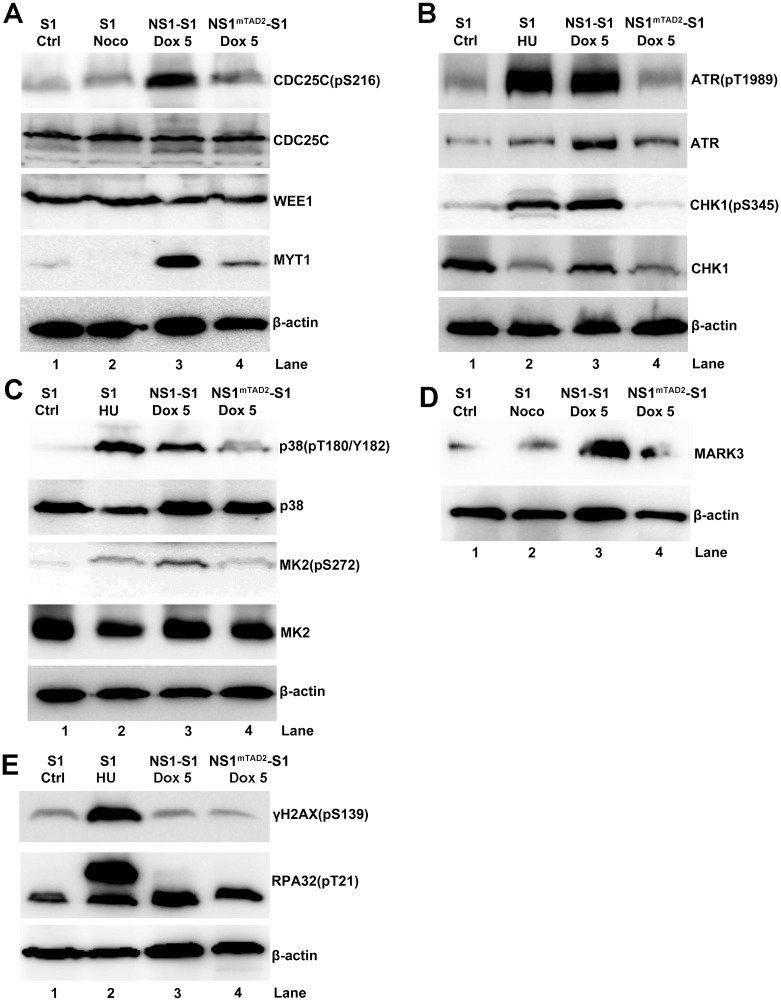
The NS1 TAD2 domain is responsible for transactivation of various cell cycle related genes. NS1-S1 and NS1^mTAD2^-S1 cells were induced by Dox (5 μg/ml) for 72 h. The cells were then collected, lysed, and immunoblotted with the indicated antibodies in each panel (A-E). S1 cells and S1 cells treated with Noco or HU were loaded as controls. β-actin was used as a loading control.

Since CDK1 is inactivated by phosphorylation at Y15, and increased phosphorylation leads to more cells arrested at G2-phase, we wanted to identify the upstream kinases that phosphorylate CDK1 at Y15. MYT1 (Myelin Transcription Factor 1) phosphorylates CDK1 at T14 and Y15 in the cytoplasm, and WEE1 phosphorylates CDK1 at Y15 in the nucleus [48–50]. In addition, CDC25C dephosphorylates CDK1 at both T14 and Y15, thereby activating CDK1 [51,52]. When we examined expression and phosphorylation of these kinases, we found that NS1-induced G2 arrest was accompanied by a significant increase in CDC25C phosphorylation at S216 (an inactive form of CDC25C) [53] (Fig 5A, lane 3 and S3A Fig), whereas expression of total CDC25C barely changed (Fig 5A, lane 3). Expression of MYT1 was upregulated in NS1-expressing cells but not in NS1^mTAD2^-expressing cells (Fig 5A, lanes 3 *vs*. 4), and expression of WEE1 was not affected under either condition (Fig 5A, WEE1). Both MYT1 and WEE1 were not upregulated in the level of mRNA in NS1-expressing cells (Fig 2A).

CDC25C plays a key role in DDR-induced cell cycle arrest, suggesting the DDR pathways (ATR-CHK1 and p38-MK2), but not ATM and its downstream kinase CHK2, inactivate CDC25C in NS1-expressing cells [54,55] (Fig 3B). Of note, we observed significant increases in ATR and ATR phosphorylated at T1989 in NS1-, but not NS1^mTAD2^-, expressing cells (Fig 5B, lanes 3 *vs*. 4, and S3B & S3C Fig). Also, the levels of phosphorylated CHK1(pS345) increased significantly in NS1-expressing cells but not in NS1^mTAD2^-expressing cells (Fig 5B, lanes 3 *vs*. 4, and S3E Fig), whereas expression of CHK1 fell in both NS1- and NS1^mTAD2^-expressing cells (Fig 5B, lanes 3 and 4, and S3D Fig). In addition, NS1 activated the p38 pathway as there was a noticeable increase in phosphorylated p38 (pT180/Y182), but not total p38, in NS1-expressing cells compared with NS1^mTAD2^-expressing cells (Fig 5C, lanes 3 *vs*. 4). We also examined MK2 (MAPKAP kinase-2) phosphorylation, which is a direct target of p38 kinase and phosphorylates CDC25C in response to UV irradiation [55]. The results showed that expression of phosphorylated MK2 (pS272), but not total MK2, increased in NS1-expressing cells (Fig 5C, lanes 3 *vs*. 4), suggesting that MK2 kinase is activated in NS1-expressing cells. MARK3 (MAP/microtubule affinity-regulating kinase 3), also called C-TAK1, regulates the cell cycle by phosphorylating CDC25C at S216 [53]. The results showed that MARK3 expression was also increased in NS1-expressing cells (Fig 5D, lanes 3 *vs*. 4), albeit it was not upregulated in the mRNA level of NS1-expressing cells (Fig 2A). HU-treated cells were used as positive controls for kinase activation (Fig 5B, 5C and 5E, lane 2).

Since ATR and p38 pathways are activated during NS1-induced G2 arrest, we asked whether NS1-mediated G2 arrest also induces a DDR. Thus, we examined expression of DDR markers γH2AX(pS139) and RPA32(pT21) in NS1-expressing cells [56–60]. Notably, and in contrast to control HU-treated cells (Fig 5E, lane 2), there was no significant difference in the levels of γH2AX(pS139) and RPA32(pT21) between NS1- and NS1^mTAD2^-expressing cells (Fig 5E, lanes 3 *vs*. 4, and S3F & S3G Fig).

Taken together, these results suggest that the DDR-signaling ATR and p38 pathways, but not the ATM pathway, are active in NS1-expressing cells, and that, surprisingly, neither pathway induces hallmarks of DDR: phosphorylation of H2AX and RPA32. Additionally, we found that MARK3 kinase was activated in NS1-expressing cells.

### ATR kinase, but not p38, MYT1, or MARK3 kinase, plays a critical role in activating the CDC25C-cyclin B1/CDK1 pathway in NS1-expressing cells

**Fig 6:**
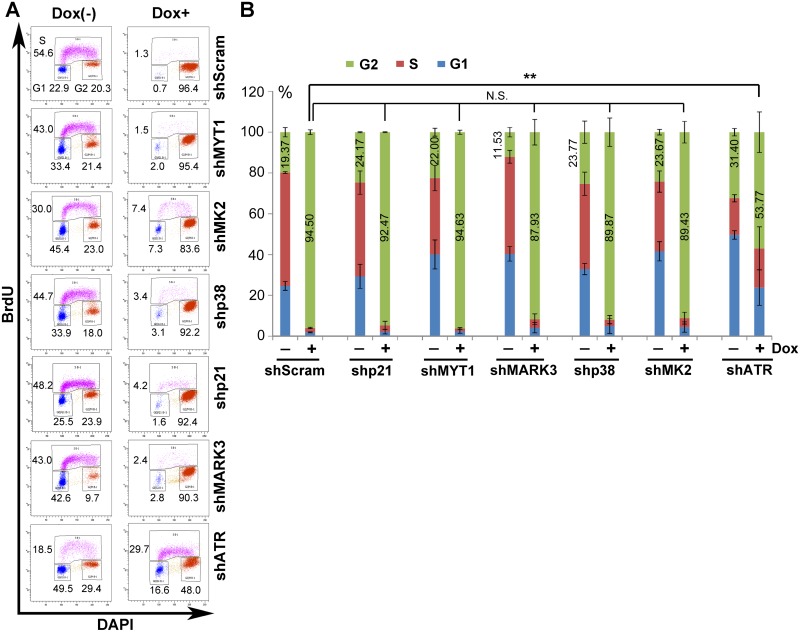
Knockdown of ATR diminishes NS1-induced G2-phase arrest in UT7/Epo-S1 cells. (A) Cell cycle analysis. NS1-S1 cells were transduced with shRNA-expressing lentivirus as indicated. After 48 h, cells were treated with Dox at 5 μg/ml (Dox+) or without (Dox-). After 72 h, the cells were collected and co-stained with an anti-BrdU antibody and DAPI. Cell cycle analysis of mCherry-expressing cells is shown. (B) Statistical analyses. The percentage of cells at G1-, S-, and G2-phase after shRNA transduction is depicted in color. The numbers show the percentages of the cells at G2-phase. The percentage of shScram and other shRNAs transduced NS1-expressing cells at G2-phase was statistically analyzed. **P<0.01, and N.S. represents no significance.

**Fig 7:**
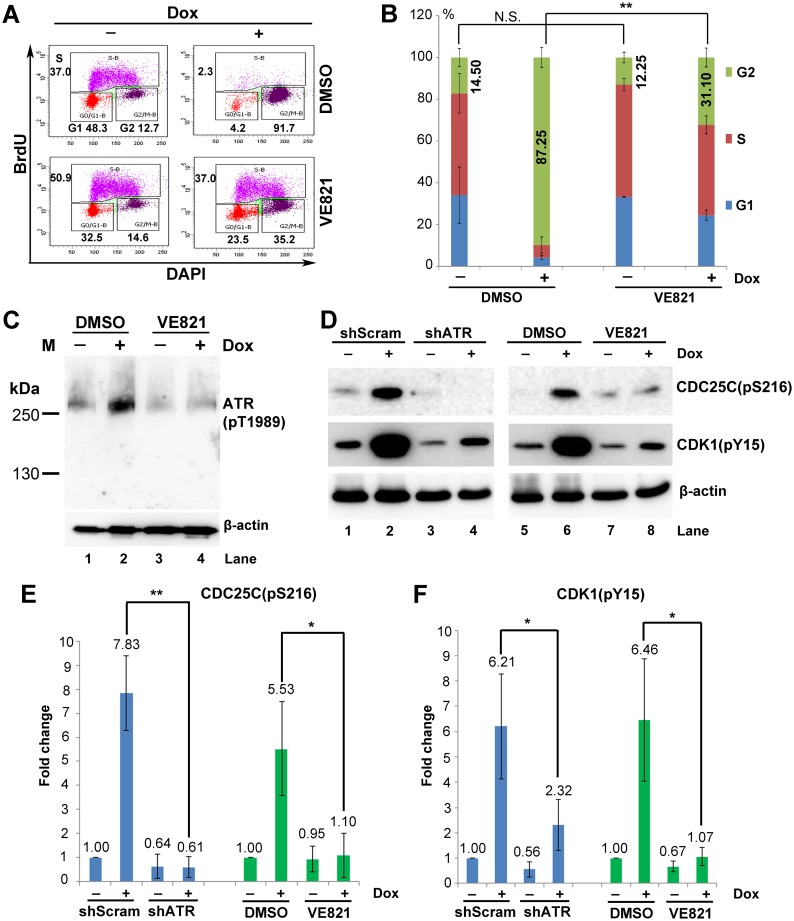
Inhibiting ATR phosphorylation abolishes NS1-induced G2-phase arrest in UT7/Epo-S1 cells. (A) Cell cycle analysis. NS1-S1 cells were treated with the ATR-specific inhibitor VE821 at 3 h prior to Dox treatment. At 72 h post-treatment, the cells were then collected and co-stained with an anti-BrdU antibody and DAPI prior for flow cytometry. DMSO-treated NS1-S1 cells were used as a control. (B) Statistical analyses. The percentage of the cells at each stage of the cell cycle is depicted in color. Numbers shown are the percentages at G2-phase and are statistically compared within cell groups treated with or without Dox induction as indicated. **P<0.01, and N.S. represents no significance. (C) ATR inhibition. After treatment with DMSO or VE821, cells were collected, and expression of ATR(pT1989) was examined by Western blotting. (D) Activation of the ATR-CDC25C-CDK1 pathway. NS1-S1 cells were either transduced with lentivirus harboring scramble or ATR-specific shRNA for 48 h, or treated with DMSO and VE821, and then treated with Dox for 72 h. The cells were then collected, lysed, and immunoblotted with the indicated antibodies. (E&F) Quantification. The detected bands of CDC25C(pS216) and CDK1(pY15) shown in panel D were quantified, and the results are expressed as the mean ± standard deviation of at least three independent experiments. Statistical analysis was performed in paired groups as indicated. **P<0.01, and *P<0.05.

Since we found that ATR-CHK1, p38-MK2, MYT1, and MARK3 kinases were activated in NS1-expressing G2-arrested cells, we next examined their contribution to NS1-induced G2 arrest. We used shRNA-expressing lentiviruses to knock down each pathway in Dox-induced NS1-expressing cells and then identified the most efficient shRNA (S4 Fig). Neither of the shp21, shMYT1, shMARK3, shp38, and shMK2 shRNAs caused a significant change in the number of the cells arrested at G2 by Dox-induced NS1 expression, compared to 94.5% of the G2-arrested cells treated with scramble shRNA (Fig 6B, N.S.). However, when cells were exposed to shRNA targeting ATR (shATR), only 53.77% of the Dox-induced cells were arrested at G2 (Fig 6B, shATR). We further confirmed the role of ATR in NS1-induced G2 arrest using an ATR-specific pharmacological inhibitor, VE821 [61]. VE821 treatment led to a significant reduction in the number of the Dox-induced cells arrested at G2 (from 87% to 31%) (Fig 7A & 7B, Dox+); there was no significant change of the cell cycle when cells were treated with VE821 in noninduced cells (Fig 7A & 7B, Dox-). As a control, VE821 effectively inhibited ATR activation in Dox-induced cells (Fig 7C, Dox+).

In NS1-expressing cells, ATR is activated by phosphorylation at T1989, CDC25C is phosphorylated at S216, and CDK1 is phosphorylated at Y15 (Fig 5). Therefore, we asked whether there was a link between these activated kinases. We knocked down ATR expression in Dox-induced cells, or inhibited its activity using VE821. As shown in Fig 7D, knockdown or inhibition of ATR led to a marked reduction in the phosphorylation of CDC25C at S216 and of CDK1 at Y15 (Fig 7E & 7F), suggesting that the ATR inactivates CDC25C, which then prevents dephosphorylation of CDK1 at Y15 and, subsequently, inactivates the cyclin B1/CDK1 complex in NS1-expressing cells.

Taken together, these results indicate that, among the kinases (ATR, p38-MK2, MYT1, and MARK3) that regulate CDC25C activation, ATR is the only one that makes a significant contribution to NS1-induced G2 arrest. In addition, although p21 was highly upregulated in NS1-expressing cells, it did not regulate NS1-induced G2 arrest (Fig 6, shp21).

### The ATR pathway plays a significant role in both B19V NS1 and infection-induced G2 arrest of CD36^+^ EPCs

**Fig 8:**
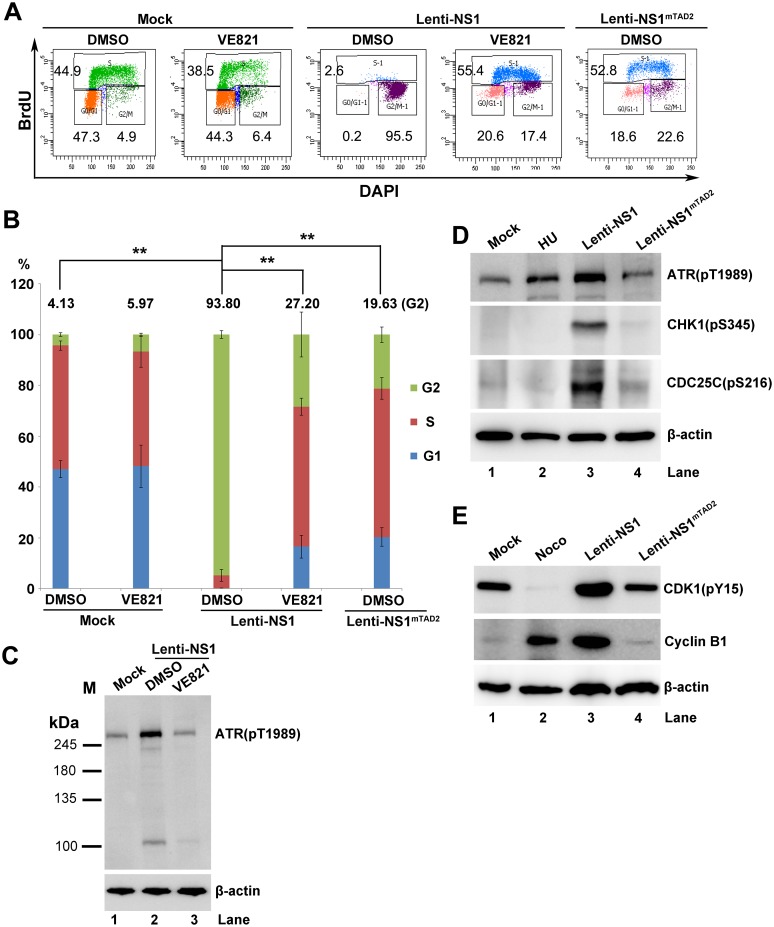
Inhibition of ATR activation inhibits the NS1-induced G2-phase arrest of CD36^+^ EPCs. (A) Cell cycle analysis. CD36^+^ EPCs were treated with VE821 at 3 h prior to NS1-expressing lentivirus transduction or mock transduction. After 48 h, cells were collected, and the cell cycle phase of NS1-expressing cells (selected by staining with anti-Flag) was examined by flow cytometry. (B) Statistical analyses. The percentage of cells treated with DMSO or VE821 that were at G1-, S-, and G2-phase is depicted in color. The numbers shown are the percentages of the cells at G2-phase, and are statistically compared as indicated. ** P<0.01. (C-E) Western blot analysis. (C) CD36^+^ EPCs were either treated with DMSO or VE821 at 3 h prior to lentivirus or mock transduction. After 48 h, cells were collected for Western blotting to detect ATR(pT1989). (D&E) CD36^+^ EPCs were transduced with Lenti-NS1 or Lenti-NS1^mTAD2^. After 48 h, cells were collected for Western blotting to detect ATR(pT1989), CHK1(pS345), CDC25C(pS216), CDK1(pY15), and β-actin. Cells treated with HU or Noco at 24 h prior to analysis were used as controls.

We next aimed to validate the role of the ATR-CHK1-CDC25C-CDK1 pathway in the B19V NS1-induced G2 arrest of CD36^+^ EPCs. NS1-expressing lentiviruses, Lenti-NS1 and Lenti-NS1^mTAD2^, were used to transduce CD36^+^ EPCs, respectively, and NS1-expressing cells were selected for cell cycle analysis. We found that 93.8% of NS1-expressing CD36^+^ EPCs were arrested at G2-phase; however, VE821-mediated inhibition of ATR led to a significant reduction (from 93.8% to 27.2%) in the percentage of cells at G2-phase (Fig 8A & 8B, Lenti-NS1). As a control, NS1^mTAD2^-expressing CD36^+^ EPCs only had 19.6% of the cells at G2-phase (Fig 8A & 8B, Lenti-NS1^mTAD2^). The difference of the cell populations in G2-phase between mock and NS1^mTAD2^-expressing CD36^+^ EPCs (4.1% *vs*. 19.6%) was likely because of the lentiviral transduction. ATR inhibition was confirmed by Western blot analysis of VE821-treated cells (Fig 8C, lanes 2 *vs*. 3). Notably, in NS1-expressing CD36^+^ EPCs, ATR was obviously phosphorylated at T1989, CHK1 at S345, CDC25C at S216, and CDK1 at Y15, together with an increase in cyclin B1 expression, compared to their levels in NS1^mTAD2^-expressing cells or mock-treated cells (Fig 8D & 8E). These results confirmed that the ATR activation plays an important role in the G2 arrest of CD36^+^ EPCs expressing B19V NS1, which is mediated by the TAD2 domain of the NS1 protein, and that the ATR-CHK1-CDC25C-CDK1 pathway is activated in the cells arrested at G2-phase.

**Fig 9:**
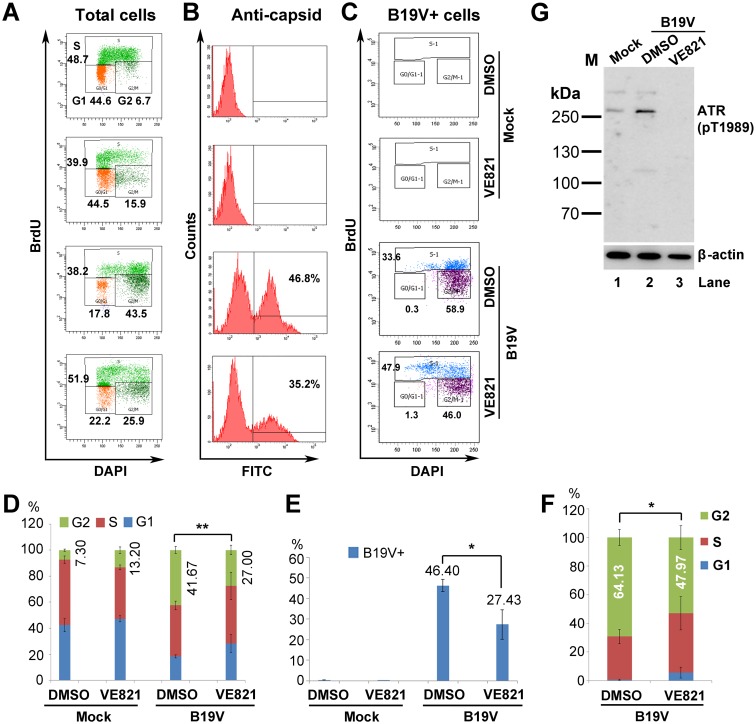
Inhibition of ATR activation significantly decreases B19V-induced G2 arrest in infected CD36^+^ EPCs. (A–C) B19V infection and cell cycle analysis. CD36^+^ EPCs were treated with VE821 for 3 h prior to B19V or mock infection. After 48 h, cells were collected, stained with an anti-capsid antibody, and cell cycle phase was examined by flow cytometry. (A) Total cells were selected for cell cycle analysis. (B) Percentage of B19V capsid-expressing cells were analyzed. (C) Anti-capsid staining-positive were selected for cell cycle analysis. (D–F) Statistical analyses. (D) The percentage of cells treated with DMSO or VE821 that were at G1-, S-, and G2-phase is depicted in color. The numbers shown are the percentages of the cells at G2, and are statistically compared as indicated. (E) The percentage of anti-capsid positive (B19V+) cells is shown the mean ± standard deviation of at least three independent experiments. (F) The percentage of capsid-expressing cells at G1-, S-, and G2-phase is depicted in color. The numbers shown are percentages of the cells at G2. **P<0.01 and *P<0.05. (G) Western blot analysis. CD36^+^ EPCs were either treated with DMSO or VE821 at 3 h prior to B19V infection or mock-infection. After 48 h, cells were collected for Western blotting to detect ATR(pT1989).

During B19V infection of CD36^+^ EPCs at an MOI of 1,000 viral genomic copies (vgc)/cell (S5 Fig), we have previously demonstrated that viral DNA replication contributes minimally to BrdU incorporation [37]. We thus used the BrdU incorporation assay to examine G2 arrest in B19V-infected CD36^+^ EPCs. We found that 41.6% of the infected CD36^+^ EPCs were arrested at G2-phase; however, VE821-mediated inhibition of ATR led to a significant reduction (from 41.6% to 27.0%) in the percentage of cells at G2-phase (Fig 9A & 9D, B19V). In addition, 46.4% of cells were infected with B19V in the absence of VE821, whereas only 27.4% of cells were infected in its presence (Fig 9B & 9E). This confirms that ATR facilitates B19V infection [39]. When we selected B19V-infected (i.e., viral capsid-expressing) cells, we found that almost all capsid-positive cells contained 4N DNA, of which 27.9% were at S-phase and 64.1% were at G2-phase (Fig 9C & 9F). Treatment with VE821 increased the percentage of cells at S-phase (to 37.5%) and reduced the percentage (to 47.9%) at G2-phase. ATR inhibition was confirmed by Western blot analysis of VE821-treated cells (Fig 9G).

**Fig 10:**
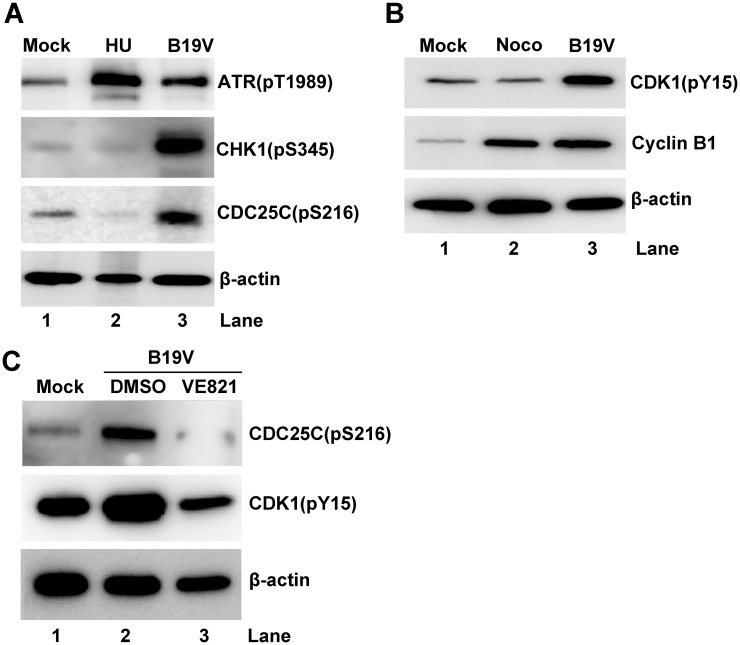
The ATR-CHK1-CDC25C-cyclin B1/CDK1 pathway is activated in B19V-infected CD36^+^ EPCs. (A&B) CD36^+^ EPCs were infected with B19V or mock-infected. (C) CD36^+^ EPCs were treated with VE821 or DMSO at 3 h prior to B19V infection. After 48 h, cells were collected, lysed, and examined by Western blot analysis for the indicated proteins. β-actin was used as a loading control.

We then further examined the ATR-CHK1-CDC25C-CDK1 pathway in B19V-infected CD36^+^ EPCs. We confirmed significant phosphorylation of ATR at T1989, of CHK1 at S345, and of CDC25C at S216 in infected cells compared with mock-infected cells (Fig 10A, and S6 Fig). Also, expression of cyclin B1 and CDK1(pY15) was significantly higher in B19V-infected EPCs than in mock-infected cells (Fig 10B and S6 Fig). These results clearly demonstrate that B19V infection of CD36^+^ EPCs activates the ATR and CDC25C pathways, and that CDK1(Y15) is not efficiently dephosphorylated. Also, expression of cyclin B1 increased significantly in B19V-infected EPCs, suggesting that the cyclin B1/CDK1 complex is inactive. Importantly, by application of VE821 in B19V-infected CD36^+^ EPCs, both CDC25C and CDK1 phosphorylation was remarkably reduced (Fig 10C).

Taken together, these results strongly suggest that ATR activation is largely responsible for the G2 arrest of CD36^+^ EPCs induced by NS1 expression, and contributes to B19V infection-induced the G2 arrest of CD36^+^ EPCs, more importantly, that the ATR-CHK1-CDC25C-CDK1 pathway is activated both in lentivirally NS1-expressing CD36^+^ EPCs and during B19V infection of CD36^+^ EPCs.

## Discussion

**Fig 11:**
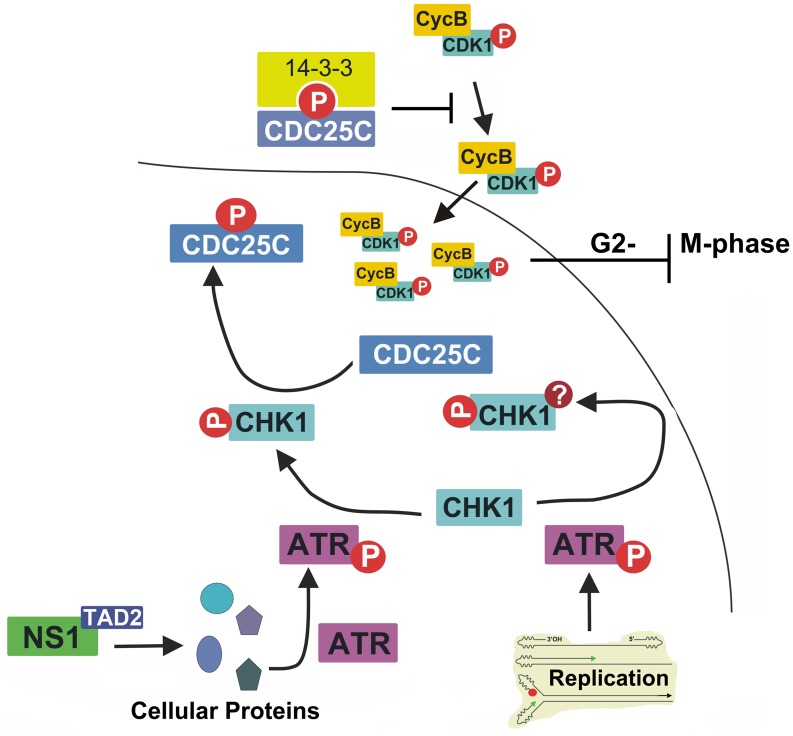
Proposed model for B19V NS1-induced G2-phase arrest. NS1 activates ATR through its TAD domain. Activated ATR then transduces signals to CDC25C through activating CHK1. CDC25C phosphatase activity is negatively regulated by phosphorylation at serine 216, which then creates a binding site for the 14-3-3 protein in the cytoplasm [41]. Thus, inactive CDC25C cannot dephosphorylate the cyclin B1/CDK1(pT14/Y15) complex; the latter component is inactive, and so progression from G2- to M-phase is blocked. It is proposed that DNA replication-induced DDR, and thereafter ATR/CHK1 activation, which plays a role in viral DNA replication, should not be involved in CDC25C phosphorylation (see the Discussion section for further explanation). The question mark indicates potential modification of CHK1, in addition to the phosphorylation at S345.

Here, we identified the key mechanism underlying B19V NS1-induced G2-phase arrest in NS1-expressing B19V-permissive cells. We found that the TAD2 domain at the C-terminus of NS1 plays a key role in transactivating cellular gene expression, and that it is essential for NS1-induced G2-phase arrest. We also identified a *de novo* role for NS1: activation of ATR. NS1 expression results in ATR phosphorylation at threonine 1989; this then inactivates CDC25C via phosphorylation at serine 216. Phosphorylated CDC25C prevents dephosphorylation of CDK1 at tyrosine15; thus, an inactive cyclin B1/CDK1 complex is imported to the nucleus, thereby blocking progression from G2- to M-phase (Fig 11). Finally, we showed that the ATR-CHK1-CDC25C-CDK1 pathway is activated in both NS1(alone)-expressing and B19V-infected CD36^+^ EPCs.

### Mechanism underlying B19V infection-induced G2-phase arrest

B19V infection of CD36^+^ EPCs and UT7/Epo-S1 cells induces cell cycle arrest of cells containing 4N DNA at “G2-phase” [12,35]. However, some infected “G2” cells containing 4N DNA also incorporated BrdU, indicating that B19V infection also induces cell cycle arrest at late S-phase [37]. Arrested CD36^+^ EPCs gradually switched from early S-phase (BrdU^+^/DNA^2N^) at the time of early infection, to late S-phase (BrdU^+^/DNA^4N^) and G2-phase (BrdU^−^/DNA^4N^) at the time of late infection. However, expression of B19V NS1 alone induces “true” G2-phase arrest (BrdU^−^/DNA^4N^), characterized by 4N DNA content without BrdU uptake [37].

Multiple mechanisms underlying B19V infection- and NS1-induced “G2-phase” (BrdU^±^/DNA^4N^) arrest have been reported. Blockade of nuclear import of the activated cyclin B1/CDK1 complex is thought to be the cause of “G2-phase” arrest in B19V-infected UT7/Epo-S1 cells [35]. By contrast, our results clearly demonstrated this is not the case. Here, the cyclin B1/CDK1 complex was efficiently imported into the nucleus. However, CDK1 within the complex remains phosphorylated at tyrosine 15. CDK1(pY15) is an inactive form of CDK1 [41]; therefore, it cannot execute its function, which is to progress the cells to M-phase. In CD36^+^ EPCs, interaction between NS1 and E2F transcription factors (E2F4/E2F5) is thought to facilitate nuclear import of E2F4/E2F5, which induces stable “G2-phase” arrest [23]. Here, we confirmed our previous results showing that the NS1 TAD2 domain is largely responsible for “true” G2-phase (BrdU^−^/DNA^4N^) arrest both in UT7/Epo-S1 cells and CD36^+^ EPCs [36], arguing the role of the nuclear import domain of NS1 in transporting E2F4/E2F5 and, thereafter, “G2-phase” arrest [23]. The specificity of the TAD2 domain in inducing G2-phase arrest through the ATR-CHK1-CDC25C-CDK1 pathway was substantiated with the detection of phosphorylated proteins in this pathway in NS1^mTAD1^ or NS1^mTAD3^-expressing UT7/Epo-S1 cells that were arrested at G2-phase to various levels (S8B–S8E Fig). In addition, comparing gene expression in NS1- *vs*. NS1^mTAD2^-expressing cells revealed that 74 genes associated with the cell cycle regulation pathway showed a ≥ 1.5-fold change in expression according to Ingenuity Pathway Analysis; only a few of these genes overlapped with E2F-regulated cell cycle genes [23]. Nevertheless, this discrepancy warrants further investigation. B19V NS1 induces cell cycle arrest at G1-phase in NS1-expressing UT7/Epo-S1 cells, which is mediated by increased expression of p21 [38]. We did not observe G1-phase arrest in NS1- or NS1^mTAD2^-expressing UT7/Epo-S1 cells (Fig 1B) or in B19V-infected CD36^+^ EPCs (Fig 8A), suggesting that neither NS1 nor B19V infection induces G1-phase arrest, although we did observe increased levels of p21 (Fig 3C).

Of note, a 5’-GTTTTGT-3’ sequence in the P6 promoter, a CpG oligodoxynucleotide-2006 analog that is a ligand for toll-like receptor 9 (TLR9), inhibits the growth of BFU-erythroid progenitors by arresting cells at the S- and G2/M-phases [62]. Thus, the viral genome is also capable of inducing cell cycle arrest of B19V-infected cells. Our previous study indicates that B19V infection-induced DDR arrests infected cells at S-phase [37]. Therefore, during infection of CD36^+^ EPCs, B19V employs multiple viral factors and cellular pathways to regulate the cell cycle. For G2-phase arrest, NS1 and the CpG oligodeoxynucleotide 2006 likely function synergistically to interrupt erythropoiesis. On the other hand, B19V infection-induced DDR, perhaps together with CpG oligodeoxynucleotide 2006, arrests infected cells at S-phase to allow efficient replication of viral DNA; S-phase factors are required for B19V DNA replication [37].

Taken together, B19V infection-induced late S-phase arrest is likely a compromise between the genome replication-induced S-phase arrest, and NS1-induced G2-phase arrest. While S-phase arrest enriches S-phase factors that favor replication of viral DNA, G2-phase arrest halts erythropoiesis in erythroid progenitors and eventually kills the cells.

### Two pathways are involved in activating ATR during B19V infection

We believe that the most striking finding of this study is that NS1 *per se* is able to activate ATR, and that ATR activation does not transduce signaling to cause phosphorylation of H2AX and RPA32, both hallmarks of a DDR [56–60]. Instead, ATR transduces signaling to inactivate CDK1 by phosphorylating CDC25C. In fact, both B19V infection and transfection of a TAD2 knockout B19V infectious clone (M20^mTAD2^) induce both a DDR and phosphorylation of H2AX and RPA32 [37]. Thus, we believe that DNA replication-activated ATR should function differently from NS1-activated ATR because, unlike TAD2, it recognizes various aberrant replicative intermediates. Interestingly, DDR (DNA replication)-induced ATR activation alone arrests the cell cycle at late S-phase, not G2-phase, in M20^TAD2^-transfected cells [37]. Therefore, we hypothesize that the function and modification (e.g. phosphorylation) of ATR activated by NS1 TAD2 are likely very different from those activated by viral DNA replication-induced DDR (Fig 11). Phosphorylation of ATR at threonine 1989 yields a functional site that transduces signals to ATR substrates, including CHK1 [63]. Further studies should examine how ATR responds to (senses) upstream stimuli.

### B19V NS1 transactivates expression of cellular genes via the TAD2 domain

B19V NS1 transactivates several human genes [30–32] as well as the viral P6 promoter [28,29]. NS1 transactivates p21 by interaction with the transcription factor Sp1 [32]. The P6 promoter contains three Sp1-binding sites (S7A Fig). Accordingly, a truncated P6 promoter, AGC/P6, which does not have a NS1-binding site (NSBS), was still transactivated by wild-type NS1, NS1^mTAD1^, and NS1^mTAD3^, but not by NS1^mTAD2^ (S7 and S8 Figs), supporting the notion that NS1 transactivates cellular genes via the TAD2 domain and Sp1. It is conceivable that many cellular genes are transcribed by CpG island promoters, which are bound mainly by Sp1 [64]. Here, we did not identify the cellular factors that interact with the TAD2 domain of NS1; this requires further investigation.

We observed that the TAD2 domain was responsible for the increased phosphorylation of ATR(pT1989) by 3.15-fold, but for the total ATR by only 1.64-fold (Fig 5B, ATR *vs*. ATR(pT1989), and S3B & S3C Fig). We hypothesize that other genes (pathways) may also contribute to ATR activation (phosphorylation). For example, transcription factor STAT5B activates the ATR/CHK1 pathway during HPV infection [65]. It is likely that NS1 transactivates other cellular genes that then activate ATR/CHK1 during B19V infection. However, STAT5B expression in UT7/Epo-S1 cells was not affected by NS1 TAD2. Although the TAD1 and TAD3 domains of B19V NS1 function to transactivate B19V P6 promoter-driven GFP expression (S8A Fig), NS1^mTAD1^ and NS1^mTAD3^ expression remained phosphorylation of ATR, CHK1 and CDC25C, which confers inactivation of the cyclin B1/CDK1 complex and thereafter G2-phase arrest (S8B–S8E Fig). Further gene expression analyses of the NS1^mTAD1^- and NS1^mTAD3^-expressing UT7/Epo-S1 cells, in comparison with NS1^mTAD2^- expressing UT7/Epo-S1 cells, would narrow down the genes that are involved in ATR activation by NS1. A ChIP-seq experiment using anti-NS1 antibody will reveal genes which are specially activated by NS1.

ATR knockdown or inhibition did not completely rescue G2-phase arrest induced by NS1 or B19V infection, although the TAD2 domain was fully responsible for NS1-induced G2 arrest in UT7/Epo-S1 cells (Fig 1). We believe that there are other pathways (in addition to the ATR-CHK1) that are involved in NS1 TAD-induced G2-phase arrest, possibly the PLK-CDC25C/CDK1 pathway. We ruled out any involvement of the p38-MK2, MYT1, and MARK3 kinases in NS1-induced G2-phase arrest. However, PLK1 kinase phosphorylates CDC25C, which then activates CDC25C [66,67].

## Materials and methods

### Ethics statement

Primary CD34^+^ hematopoietic stem cells were isolated from bone marrow of healthy human donors. We purchased the cells at AllCells LLC. (www.allcells.com, Alameda, CA), which were all anonymized, and, therefore, an IRB review was waived.

### Primary cells and cell lines

#### Primary human CD36^+^ Erythroid Progenitor Cells (CD36^+^ EPCs)

CD34^+^ hematopoietic stem cells were ex vivo expanded under normoxic conditions (21% O_2_; 5% CO_2_) at 37°C until Day 4 (the day of procurement was designated Day 0). On Day 4, the expanded cells were frozen in liquid nitrogen and labeled “Day 4 cells”. For each experiment, Day 4 cells were thawed and cultured for 2–3 days in expansion Wong medium [68,69] under normoxic conditions. On Day 6 or 7, cells were cultured under hypoxic conditions (1% O_2_) for 2 days; these cells expressed CD36 (a marker for erythroid cells) and so were termed CD36^+^ erythroid progenitor cells (CD36^+^ EPCs) [69].

#### UT7/Epo-S1 cells

UT7/Epo-S1 cells, a megakaryoblastoid cell line [35], were cultured under normoxic conditions in Dulbecco’s modified Eagle’s medium containing 10% fetal bovine serum and erythropoietin (2 U/ml; Amgen, Thousand Oaks, CA).

#### NS1-S1, NS1^mTAD1^-S1, NS1^mTAD2^-S1, NS1^mTAD3^-S1 cell lines

UT7/Epo-S1 cells were transduced with a TripZ lentiviral vector expressing doxycycline (Dox)-inducible NS1, NS1^mTAD1^, NS1^mTAD2^, or NS1^mTAD3^ (see below). At 48 h post-transduction, puromycin was added at a concentration of 0.5 μg/ml and the cells were cultured for 3–4 passages to select those that were puromycin-resistant. Dox was then added (final concentration, 5 μg/ml) to induce NS1 expression.

### Infection with B19V

Plasma samples containing B19V were obtained from Viracor-IBT Laboratories (Lee’s Summit, MO). The virus titer was measured and expressed as viral genomic copies per ml (vgc/ml), as reported previously [70]. Sample no. P404 (~1 × 10^12^ vgc/ml) was used for all infection experiments. CD36^+^ EPCs were infected with B19V at a multiplicity of infection (MOI) of ~1,000 vgc/cell.

### Lentiviral vector construction

#### pTripZ vectors

The codon-optimized B19V NS1-coding sequence (opt-NS1) has been described previously [36]. The opt-NS1^mTAD1^, opt-NS1^mTAD2^, opt-NS1^mTAD3^ mutants were constructed by the introduction of four alanine mutations, ^416^GAIAFVVAA^424^, ^523^AAFFALIAP^531^, ^566^AAWAAAFAT^574^, into the putative TAD1, TAD2, and TAD3 domains of B19V NS1 [36], respectively. The pTripZ-NS1-Strep-Flag, pTripZ-NS1^mTAD1^-Strep-Flag, pTripZ-NS1^mTAD2^-Strep-Flag, and pTripZ-NS1^mTAD3^-Strep-Flag vectors were constructed by inserting C-terminally StrepII-3×Flag-tagged opt-NS1, opt-NS1^mTAD1^, opt-NS1^mTAD2^, and opt-NS1^mTAD3^ coding sequences, respectively, into the pTripZ vector (GE Dharmacon, Lafayette, CO) via the *Age I* and *Mlu I* restriction sites.

#### pLenti vectors

pLenti-NS1 (pLenti-½ITR-P6-NS1) and pLenti-NS1^mTAD2^ [pLenti-½ITR-P6-NS1(mTAD2)] have been described previously, in which the NS1 is C-terminally Flag tagged [36]. pLenti-ATF/p6-GFP and pLenti-AGC/p6-GFP were constructed by inserting different sized B19V P6 promoter-driven GFP cDNA fragments into the pLenti-GFP vector [69] via the *Cla I* and *Xba I* sites.

#### pLKO vectors

Lentiviral vector pLKO.1-mCherry was used to clone shRNA sequences between the *Age I* and *EcoRI* sites, as described previously [71]. Vector pLKO.1-mCherry containing scramble shRNA was used as control for each experiment [69]. Validated MYT1, MAPKAPK2 (MK2), p38MAPK (p38), p21, MARK3, and ATR shRNAs were obtained from Sigma (St. Louis, MO). All the sequences of shRNAs used are listed in S1 Table.

### Lentivirus production and transduction

The production and concentration of lentiviruses were performed according to the instructions provided by Addgene (http://www.addgene.org/plko). Cells were transduced with lentiviral vector at a MOI of 2–4 transduction units/cell, as described previously [69].

### RNA isolation and RNA-seq analysis

#### RNA isolation

NS1-S1 and NS1^mTAD2^-S1 cells were treated with Dox (Dox+; 5 μg/ml) or without (Dox-) for 72 h, after which they were collected and washed with phosphate buffered saline (PBS; pH 7.4), suspended in RNAlater RNA Stabilization Solution (Invitrogen), and maintained at 4°C for the next step. An equal volume of ice-cold PBS was then added to dilute the RNAlater solution before centrifugation. The cells were then centrifuged at 500 g for 5 min, and the supernatant was discarded prior to addition of TRIzol reagent (Invitrogen, Carlsbad, CA) to extract total RNA. This procedure was repeated three times. At least two RNA samples were prepared from each cell type and analyzed on an Agilent 2100 Bioanalyzer with an Agilent RNA 6000 Nano Kit. RNA samples with an RNA Integrity Number (RIN) > 8.0 were used to construct cDNA libraries for RNA-seq.

#### RNA-seq

RNA-seq analysis of total RNA was performed at the Department of Biotechnology, Beijing Institute of Radiation Medicine, Beijing, China. For each sample, the NEBNext Ultra RNA library Prep Kit (Catalog# E7530L, NEB, Ipswich, MA) was used to prepare a sequencing library from 1 μg of total RNA, and 2 × 100 paired-end sequencing in high output run mode was performed using the HiSeq 2500 and cBot instrument, TruSeq PE Cluster Kit v3-cBot-HS, and Illumina TruSeq SBS Kit v3-HS (200 Cycles). Three RNA samples from each group (different cell lines treated with/without Dox) were subjected to RNA-seq.

#### Bioinformatics analysis

The resulting base calling (.bcl) files were converted to FASTQ files using Illumina’s bcl2fastq v2.17.1.14 software. Mapping RNA-seq reads on the human genome (GRCh38) after trimming the adaptors, transcript assembly, and abundance estimation were performed using Tuxedo Suite pipeline (TopHat v2.0.9/Cufflinks v2.2.1) [72,73] and reported using Fragments Per Kilobase of exon per Million fragments mapped (FPKM). Cuffdiff was used to calculate statistically significant changes in gene expression between the NS1-S1 and NS1^mTAD2^-S1 cell lines with/without Dox induction.

To identify the cellular pathways in which differentially expressed genes function, DAVID (the Database for Annotation, Visualization and Integrated Discovery) Bioinformatics Resources (https://david.ncifcrf.gov/) [74] was used to perform Kyoto Encyclopedia of Genes and Genomes (KEGG) pathway analysis [75]. Briefly, genes showing a significant change (Q value < 0.05 and -fold change ≥ 1.5) in expression between NS1-S1/Dox+ and NS1^mTAD2^-S1/Dox+ were loaded into the DAVID Bioinformatics Resources.

### BrdU incorporation assay and flow cytometry

*De novo* DNA synthesis was tracked using a BrdU incorporation assay, as previously described [37]. Briefly, cells were collected and incubated with BrdU at 30 μM for 1 h. After BrdU incorporation, cells were collected, fixed and permeabilized. Then, the cells were incubated with 1N HCl for 30 min. BrdU-labeled DNA was detected using an anti-BrdU monoclonal antibody (clone B44) or a rabbit anti-BrdU polyclonal antibody. A mouse anti-B19V capsid monoclonal antibody (clone 521-5D) was used to detect B19V-infected cells, and a mouse anti-Flag monoclonal antibody was used to immunostain lentivirally Flag-tagged NS1-transduced CD36^+^ EPCs, followed by the corresponding secondary antibodies. Cells were stained with DAPI (4’,6-diamino-2-phenylindole), prior to flow cytometry analysis on the 3-laser flow cytometer (LSR II; BD Biosciences, San Jose, CA) to examine cell cycle progression, and data were analyzed using FACS DIVA software (BD Biosciences).

### Preparation of whole cell lysates and nuclear extracts

Cells stably expressing NS1 and NS1^mTAD2^ were collected at 72 h post-Dox induction and washed with ice-cold PBS. Whole cell extracts were prepared by resuspending the cells in ice-cold Cell Lysis Buffer (50 mM HEPES, pH 7.0, 250 mM NaCl, 0.1% NP-40, 10% glycerol, 1 mM DTT, and 1 mM PMSF) [35]. For nuclear extracts [76], collected cells were washed with PBS, pelleted, resuspended in 5 × package cell volumes of Lysis Buffer (10 mM HEPES, pH 7.9, 10 mM KCl, 0.1 mM EDTA, 1 mM DTT, 0.5% NP-40, and 0.5 mM PMSF), and kept on ice for 5 min. The tubes were then vortexed gently to disrupt the cell membranes and centrifuged at 500 × g for 5 min at 4°C. The pelleted nuclei were washed twice with the Lysis Buffer without NP-40, resuspended in a 1 × package cell volume of Nuclear Extraction Buffer (20 mM HEPES, pH 7.9, 420 mM NaCl, 1 mM EDTA, 1 mM DTT, 1 mM PMSF, and 25% glycerol), and incubated on ice for 30 min. The samples were further centrifuged at 12,000 × g for 10 min at 4°C. The supernatants were collected and used as nuclear extracts.

### Co-immunoprecipitation (Co-IP) assay and *in vitro* CDK1 kinase assay

We followed a previously published *in vitro* CDK1 kinase assay [35]. The Pierce Cross-link IP kit (Thermo Fisher Scientific, Waltham, MA) was used to for Co-IP. Briefly, 4 μg of anti-cyclin B1 antibody we crosslinked to 20 μl of the protein A/G plus agarose for each sample. We diluted the whole cell extracts of 600 μg in Cell Lysis Buffer described above, and the nuclear extracts of 600 μg in Dilution Buffer (20mM HEPES, pH 7.9, 0.1mM EDTA, 1mM DTT, 1mM PMSF), to a total volume of 300 μl. Then, each sample was incubated with the antibody-crosslinked beads at 4°C overnight.

For Co-IP assay, precipitated proteins were eluted with Elution Buffer provided with the IP kit, resuspended with 5 × protein loading buffer, and boiled for Western blot analysis. For kinase assay, the beads were then washed with Lysis Buffer or Nuclear Extraction Buffer, and then incubated with 20 μl of Reaction Buffer (20 mM HEPES, pH 7.9, 5 mM MgCl_2_, 1 μg of histone H1, 1 mM DTT, 100 μM unlabeled ATP, and 10 μCi of [γ-^32^P] ATP) at 37°C for 30 min to 1 h. The reaction was stopped by addition of 5 μl of 5 × protein loading buffer, followed by boiling for 5 min. An aliquot of 10 μl of each sample was loaded onto 12% SDS-polyacrylamide gels, and the samples were separated by electrophoresis. The gels were dried and subjected to autoradiography.

### Western blot analysis

Western blotting was performed as described previously [37,39,70]. Signals were developed using a Fuji LAS3000 imaging system. Band intensities were quantified using Image Quant TL 8.1 software (GE Health Life Sciences, Pittsburgh, PA).

### Statistics analysis

For cell cycle analysis, the percentage of cells at each point of the cell cycle was determined from at least three independent experiments and expressed as the mean ± standard deviation. For protein expression analysis, quantification of the protein band in each group on Western blots was normalized to the level of β-actin expression, and relative level to the band of the control group was calculated. The quantification results are expressed as the mean ± standard deviation of at least three independent experiments. P values were calculated using Student’s t test. **P<0.01 and *P<0.05 were regarded as statistically significant, and N.S. as statistically no significance.

### Antibodies used in the study

The following first antibodies were purchased: anti-Strep (A01732) from GenScript, Piscataway, NJ; mouse anti-BrdU (clone B44) (347580), anti-cyclin B1 (554179), anti-CDK1 (pY15) (612306) from BD, San Jose, CA; rabbit anti-BrdU (600-401-C29) and mouse anti-Flag (2001-301-1313) from Rockland, Limerick, PA; anti-CDK1 (A303-663A), anti-CDC25C (A301-390A), anti-MYT1 (A302-424A), anti-p38 (A300-707A) from Bethyl, Montgomery, TX; anti-CDK1 (pT161) (P12-1090) from Assay Biotech, Sunnyvale, CA; anti-CDC25C(pS216) (4204), anti-γH2AX(pS139) (05–636), anti-B19V capsid (MAB8292) from Millipore, Billerica, MA; anti-WEE1 (AP8106b), anti-CHK1 (AM7401a), anti-CHK2 (AP4999a), anti-MAPKAPK2(pS272) (AP3147a) from Abgent, San Diego, CA; anti-ATR (ab2905), anti-GAPDH (Ab8245) from Abcam, Cambridge, MA; anti-ATR (pT1989) (GTX128145), anti-CHK1(pS345) (GTX100065S), anti-RPA32(pT21) (GTX62664) from GeneTex, Irvine, CA; anti-ATM (2873), anti-MAPKAPK2(MK2) (12155s), anti-MARK3 (9311s), anti-p21 (2947p), anti-Histone H3 (9715S) from Cell Signaling Technology, Danvers, MA; anti-ATM(pS1981) (2952–1), anti-CHK2(pS33/35) (2297–1) from Epitomics, Burlingame, CA; anti-p38 (pT180/Y182) (p190-1802) from Phosphosolutions, Aurora, CO; anti-β-actin (A5441) from Sigma-Aldrich, St. Louis, MO.

Second antibodies used are HRP-conjugated anti-mouse IgG and HRP-conjugated anti-rabbit IgG from Sigma, and FITC- and Rhodamine-conjugated anti-mouse IgG and Alex Fluor 594-conjugated anti-rabbit IgG from Jackson Immuno Research Inc., West Grove, PA.

### Chemicals and pharmacological inhibitors

The ATR-specific pharmacological inhibitor VE-821 [61] (SelleckChem, Houston, TX), puromycin (Santa Cruz, Dallas, Texas), hydroxyurea (HU; Calbiochem/Millipore, Billerica, MA), Dox, nocodazole (Noco), and BrdU (Sigma, St. Louis, MO) were all purchased.

Cells were treated with Dox (5 μg/ml) and analyzed 72 h later. At 24 h prior to analysis, cells were treated with HU (10 mM) or Noco (0.5 μg/ml); these cells served as controls for DDR induction and G2 arrest, respectively. At 3 h prior to Dox induction or infection, UT7/Epo-S1 cells and CD36^+^ EPCs were treated with VE-821 at 1 or 10 μM, respectively.

## Supporting information

S1 Fig NS1 and NS1^mTAD2^ expression of Dox-induced NS1-S1 and NS1^mTAD2^-S1 cells. NS1-S1 and NS1^mTAD2^-S1 cells were treated with doxycycline (Dox) at 5 μg/ml or without [Dox(-)]. (A) Immunofluorescence analysis. At 72 h post-treatment, cells were collected, fixed, and permeablized. Then, the cells were stained with a moue anti-Flag antibody, followed by a Rhodamine-conjugated anti-mouse secondary antibody. Images were taken under a Nikon Eclipse Ti-S inverted microscope at 10 × magnification. Dox(-) cells were used as negative controls. (B) Flow cytometry analysis. At 72 h post-treatment, cells were collected, fixed, and permeablized. Then, the cells were stained with a mouse anti-Flag antibody, followed by a fluorescein isothiocyanate (FITC)-conjugated anti-mouse secondary antibody. The stained cells were analyzed for NS1 or NS1^mTAD2^ expression by flow cytometry. The percentages of NS1- or NS1^mTAD2^-expressing cells (anti-Flag positive) are indicated. Anti-Flag stained (positive) cells were gated by setting the NS1-S1/Dox(-) cells as the negative background.
(TIF)

S2 Fig Quantification of CDK1(pT161), ATM, and ATM(pS1981) expressed in NS1-S1 and NS1^mTAD2^-S1 cells. The lower band of (A) CDK1(pT161) and the bands of (B) ATM and (C) ATM(pS1981) shown in Fig 3 were quantified from at least three independently performed blots. The quantifications are expressed as the mean ± standard deviation. Statistical analysis was performed in paired groups as indicated. *P<0.05, and N.S. denotes no significant difference.
(TIF)

S3 Fig Quantification of ATR, ATR(pT1989), CHK1, CHK1(pS345), γH2AX(pS139), RPA32(pT21), and CDC25C(pS216) expressed in NS1-S1 and NS1^mTAD2^-S1 cells. The detected bands on the blots shown in Fig 5 were quantified as the expression levels of indicated proteins: (A) CDC25C(pS216), (B) ATR, (C) ATR(pT1989), (D) CHK1, (E) CHK1(pS345), (F) γH2AX(pS139), and (G) RPA32(pT21), and the results are expressed as the mean ± standard deviation of at least three independent experiments. Statistical analysis was performed in paired groups as indicated. **P<0.01, *P<0.05, and N.S. denotes no significant difference.
(TIF)

S4 Fig Efficiency of shRNA knockdown. NS1-S1 cells were transduced with shRNA-expressing lentivirus as indicated. At 48 h post-transduction, Dox was added at 5 μg/ml. After 72 h, the cells were collected for Western blot analysis of knockdown efficiency of the indicated proteins: (A) MYT1, (B) MK2, (C) p38, (D) p21, (E) MARK3, and (F) ATR(pT1989).
(TIF)

S5 Fig Immunofluorescence analysis of B19V-infected CD36^+^ EPCs. CD36^+^ EPCs were either infected with B19V or mock-infected. At 48 h post-infection, cells were fixed and stained with an anti-B19V capsid antibody. Images were obtained under a Nikon Eclipse Ti-S inverted microscope at 10 × magnification. DAPI was used to stain nucleus.
(TIF)

S6 Fig Quantification of protein expression of B19V infected CD36^+^ EPCs. The detected bands on the blots shown in Fig 10A & 10B were quantified as the expression levels of indicated proteins: (A) ATR(pT1989), (B) CHK1(pS345), (C) CDC25C(pS216), (D) CDK1(pY15), and (E) Cyclin B1, and the results are expressed as the mean ± standard deviation of at least three independent experiments. Statistical analysis was performed in paired groups as indicated. **P<0.01 and *P<0.05.
(TIF)

S7 Fig The TAD2 domain is critical for NS1 transactivation of the viral P6 promoter. (A) A diagram of the P6 promoter. B19V sequence of nt 241–531 (GenBank accession no.: AY386330) is shown. Important putative motifs on the P6 promoter are highlighted. (B&C) NS1, but not NS1^mTAD2^, transactivates the P6 promoter. UT7/Epo-S1 (S1) cells, S1 cells treated with nocodazole (S1/Noco), and NS1-S1 and NS1^mTAD2^-S1 cell lines treated with/without Dox (Dox-/Dox+) were transduced with Lenti-ATF/p6-GFP or Lenti-AGC/p6-GFP. (B) Immunofluorescence analysis. At 48 h post-transduction, cells were observed under a Nikon inverted fluorescent microscope and images were acquired at 10 × magnification. (C) Flow cytometry analysis. At 48 h post-transduction. cells were collected for flow cytometry analysis to determine the mean florescence intensity. Relative mean florescence intensity is shown as the mean ± standard deviation of at least three independent experiments. Paired groups were statistically analyzed. **P<0.01 and *P<0.05.
(TIF)

S8 Fig NS1^mTAD1^-S1 and NS1^mTAD3^-S1 cells induce G2-phase arrest through activation of the ATR-CHK1-CDC25C-CDK1 pathway. (A) B19V NS1 mutants, NS1^mTAD1^ and NS1^mTAD3^, transactivate P6 promoter. UT7/Epo-S1 (S1) cells, S1 cells treated with nocodazole (S1/Noco), and NS1^mTAD1^-S1 and NS1^mTAD3^-S1 cell lines treated with/without Dox (Dox-/Dox+) were transduced with Lenti-ATF/p6-GFP. At 48 h post-transduction, cells were collected for flow cytometry analysis to determine the mean florescence intensity. Relative mean florescence intensity is shown as the mean ± standard deviation of at least three independent experiments. Paired groups were statistically analyzed. **P<0.01 and *P<0.05. (B&C) Cell cycle analysis. (B) NS1^mTAD1^-S1 and NS1^mTAD3^-S1 cells were treated with Dox (Dox+) or not (Dox-) for 72 h, and then were analyzed for cell cycle using flow cytometry. The numbers shown in each histogram are percentages of the cell populations at G1-, S-, and G2-phase, respectively. (C) Statistical analysis. The percentage of cells at G1, S, and G2 are depicted in color. The percentages of the cells at G2 are shown in numbers, and compared in pairs as shown. *P<0.05, **P<0.01. (D&E) Analysis of the ATR-CHK1-CDC25C-CDK1 pathway. NS1^mTAD1^-S1 and NS1^mTAD3^-S1 cells were treated with Dox and collected for lysis 72 h later. (D) Cell lysates were analyzed for expression of phosphorylated ATR, ATR(pT1989), phosphorylated CHK1 and CDC25C, CHK1(pS345) and CDC25C(pS216), and NS1 (using an anti-Strep antibody) by Western blotting. (E) Cell lysates were further analyzed for expression of CDK(pY15) and cyclin B1 by Western blotting. β-actin was used as a loading control. Untreated S1 cells, S1 cells treated with nocodazole (Noco), and S1 cells treated with HU were used as controls.
(TIF)

S1 Table Sequences of various shRNAs used in the study. Nucleotide sequences used to generate MYT1, MAPKAPK2 (MK2), p38MAPK (p38), p21, MARK3, and ATR lentiviral shRNA vectors are shown from 5’ to 3’ end. Validated shRNA sequences were obtained from Sigma (St. Louis, MO).
(DOCX)
