# Supplementary figures and images for: Parvovirus B19 NS1 protein induces cell cycle arrest at G2-phase by activating the ATR-CDC25C-CDK1 pathway
Source: PLoS Pathog. 2017 Mar 6;13(3):e1006266. doi: 10.1371/journal.ppat.1006266 (PMC5354443; doi:10.1371/journal.ppat.1006266)

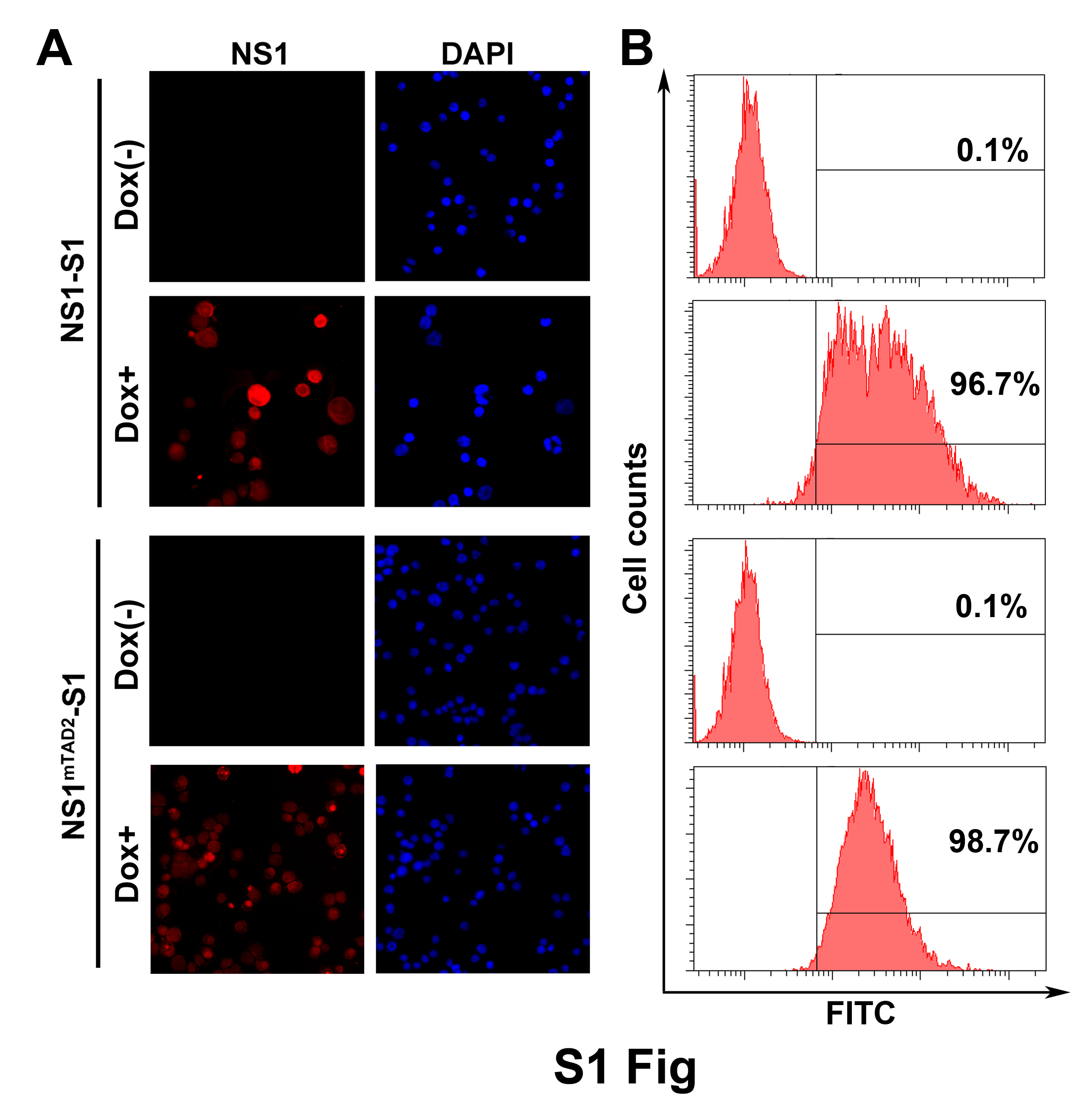

Supplement: S1 Fig — NS1-S1 and NS1mTAD2-S1 cells were treated with doxycycline (Dox) at 5 μg/ml or without [Dox(-)]. (A) Immunofluorescence analysis. At 72 h post-treatment, cells were collected, fixed, and permeablized. Then, the cells were stained with a moue anti-Flag antibody, followed by a Rhodamine-conjugated anti-mouse secondary antibody. Images were taken under a Nikon Eclipse Ti-S inverted microscope at 10 × magnification. Dox(-) cells were used as negative controls. (B) Flow cytometry analysis. At 72 h post-treatment, cells were collected, fixed, and permeablized. Then, the cells were stained with a mouse anti-Flag antibody, followed by a fluorescein isothiocyanate (FITC)-conjugated anti-mouse secondary antibody. The stained cells were analyzed for NS1 or NS1mTAD2 expression by flow cytometry. The percentages of NS1- or NS1mTAD2-expressing cells (anti-Flag positive) are indicated. Anti-Flag stained (positive) cells were gated by setting the NS1-S1/Dox(-) cells as the negative background. (TIF) [file ppat.1006266.s001.tif]

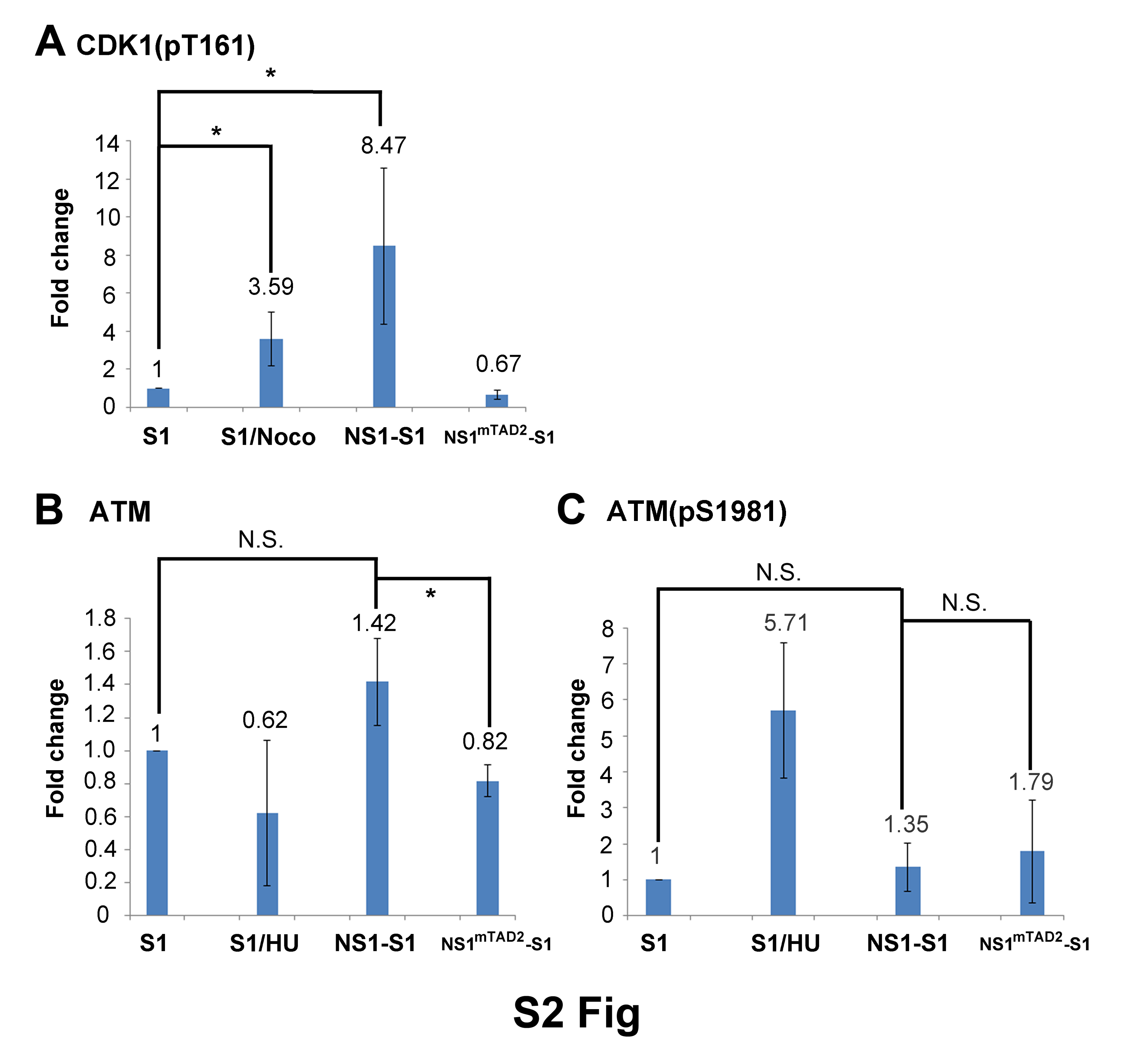

Supplement: S2 Fig — The lower band of (A) CDK1(pT161) and the bands of (B) ATM and (C) ATM(pS1981) shown in Fig 3 were quantified from at least three independently performed blots. The quantifications are expressed as the mean ± standard deviation. Statistical analysis was performed in paired groups as indicated. *P<0.05, and N.S. denotes no significant difference. (TIF) [file ppat.1006266.s002.tif]

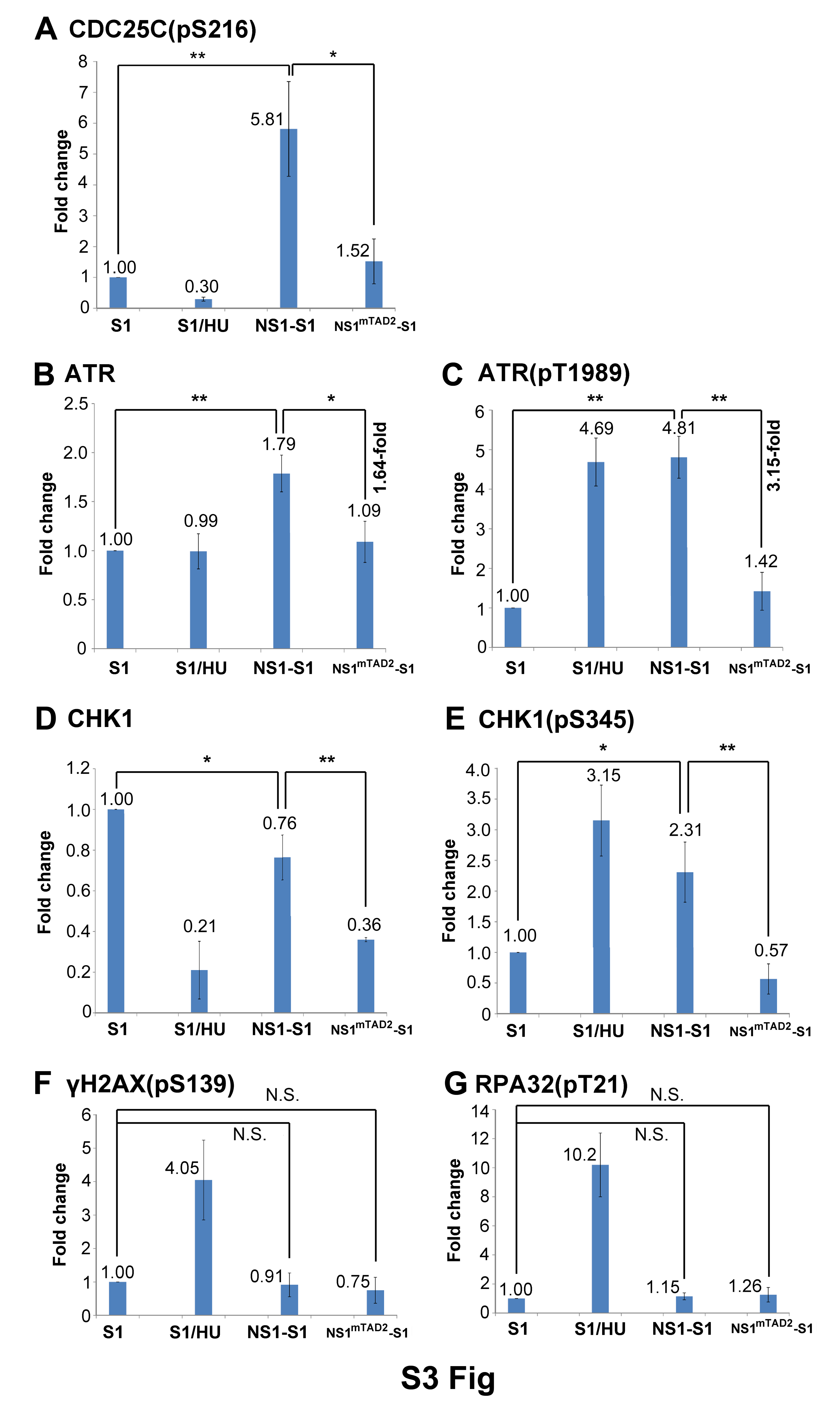

Supplement: S3 Fig — The detected bands on the blots shown in Fig 5 were quantified as the expression levels of indicated proteins: (A) CDC25C(pS216), (B) ATR, (C) ATR(pT1989), (D) CHK1, (E) CHK1(pS345), (F) γH2AX(pS139), and (G) RPA32(pT21), and the results are expressed as the mean ± standard deviation of at least three independent experiments. Statistical analysis was performed in paired groups as indicated. **P<0.01, *P<0.05, and N.S. denotes no significant difference. (TIF) [file ppat.1006266.s003.tif]

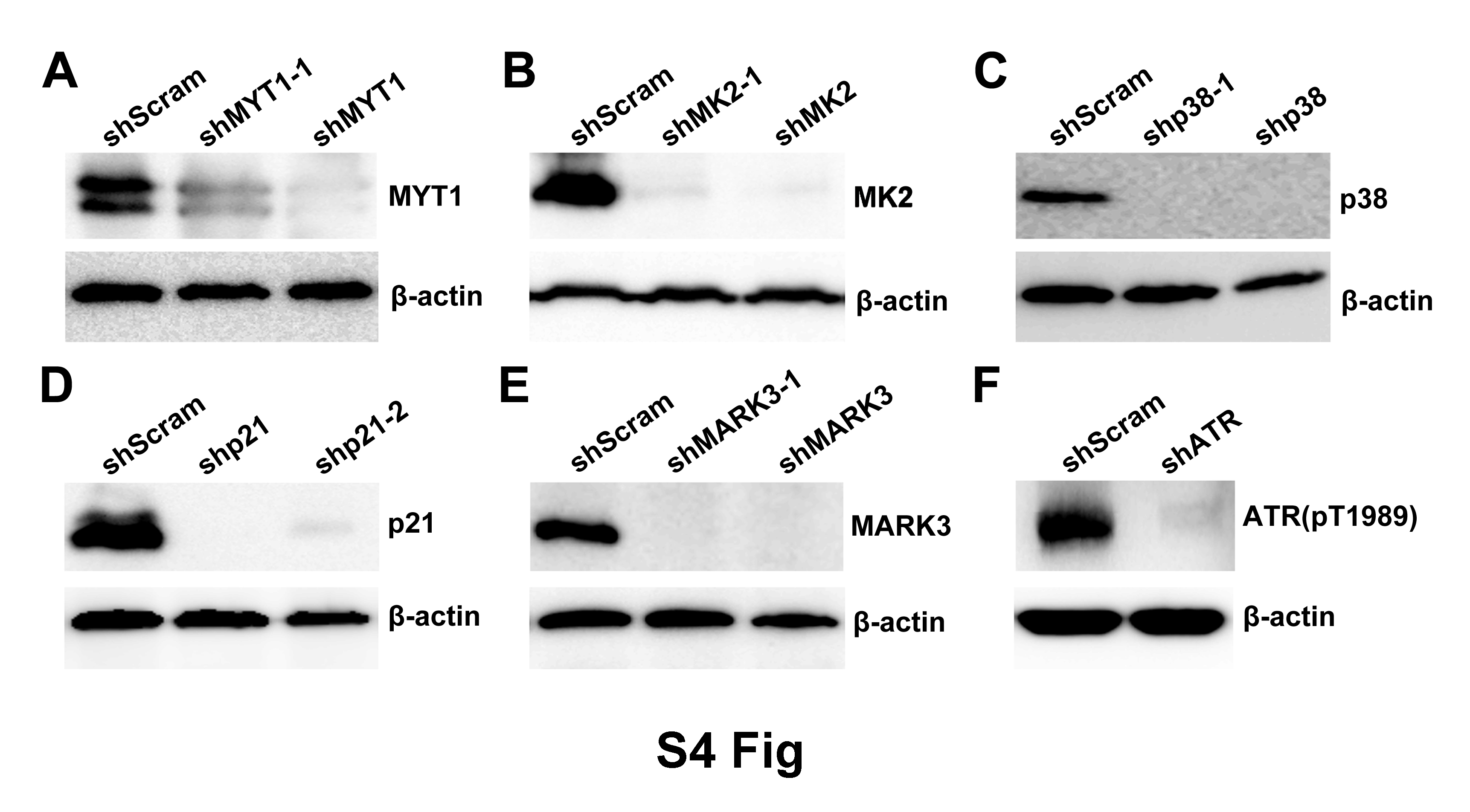

Supplement: S4 Fig — NS1-S1 cells were transduced with shRNA-expressing lentivirus as indicated. At 48 h post-transduction, Dox was added at 5 μg/ml. After 72 h, the cells were collected for Western blot analysis of knockdown efficiency of the indicated proteins: (A) MYT1, (B) MK2, (C) p38, (D) p21, (E) MARK3, and (F) ATR(pT1989). (TIF) [file ppat.1006266.s004.tif]

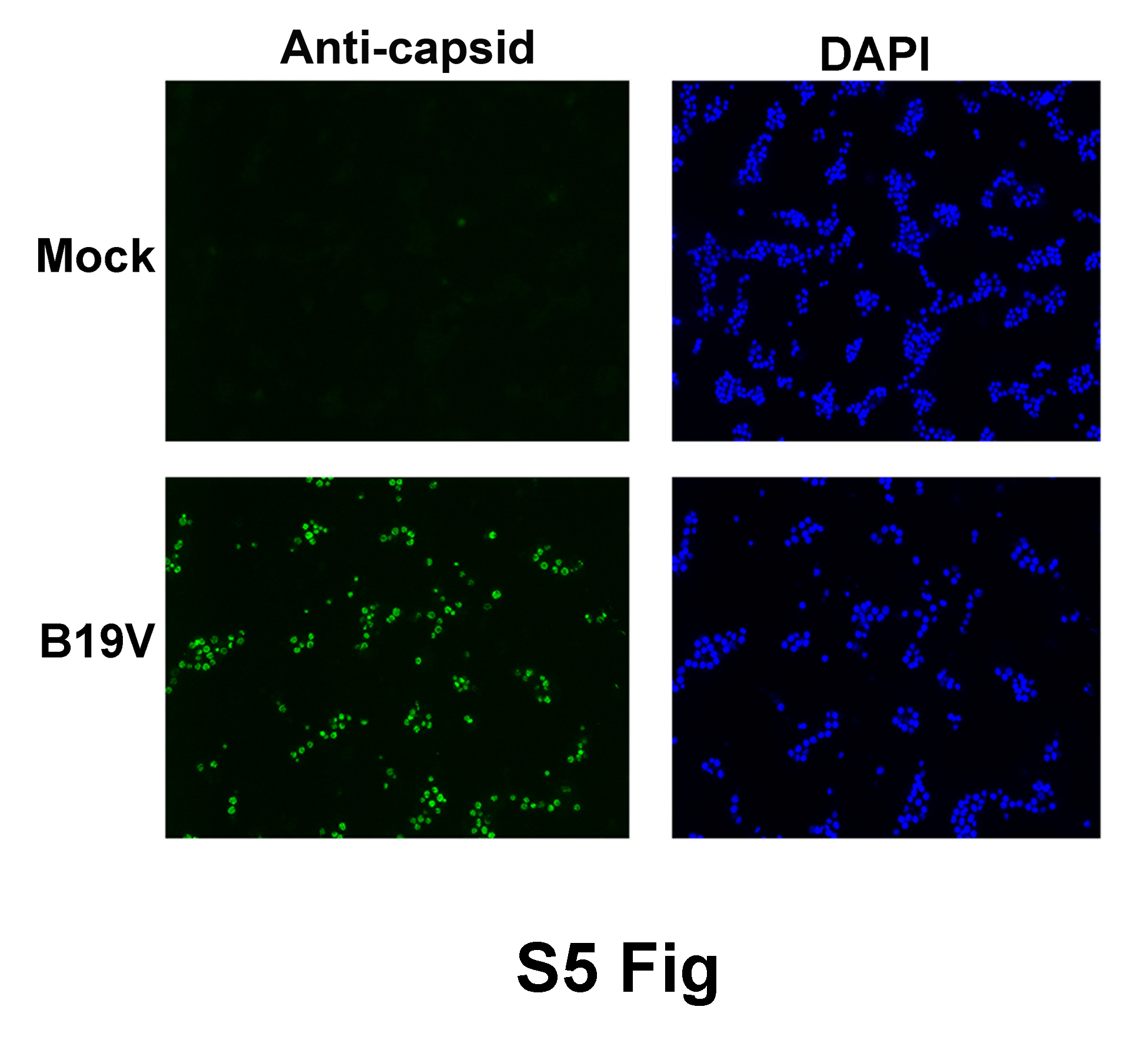

Supplement: S5 Fig — CD36+ EPCs were either infected with B19V or mock-infected. At 48 h post-infection, cells were fixed and stained with an anti-B19V capsid antibody. Images were obtained under a Nikon Eclipse Ti-S inverted microscope at 10 × magnification. DAPI was used to stain nucleus. (TIF) [file ppat.1006266.s005.tif]

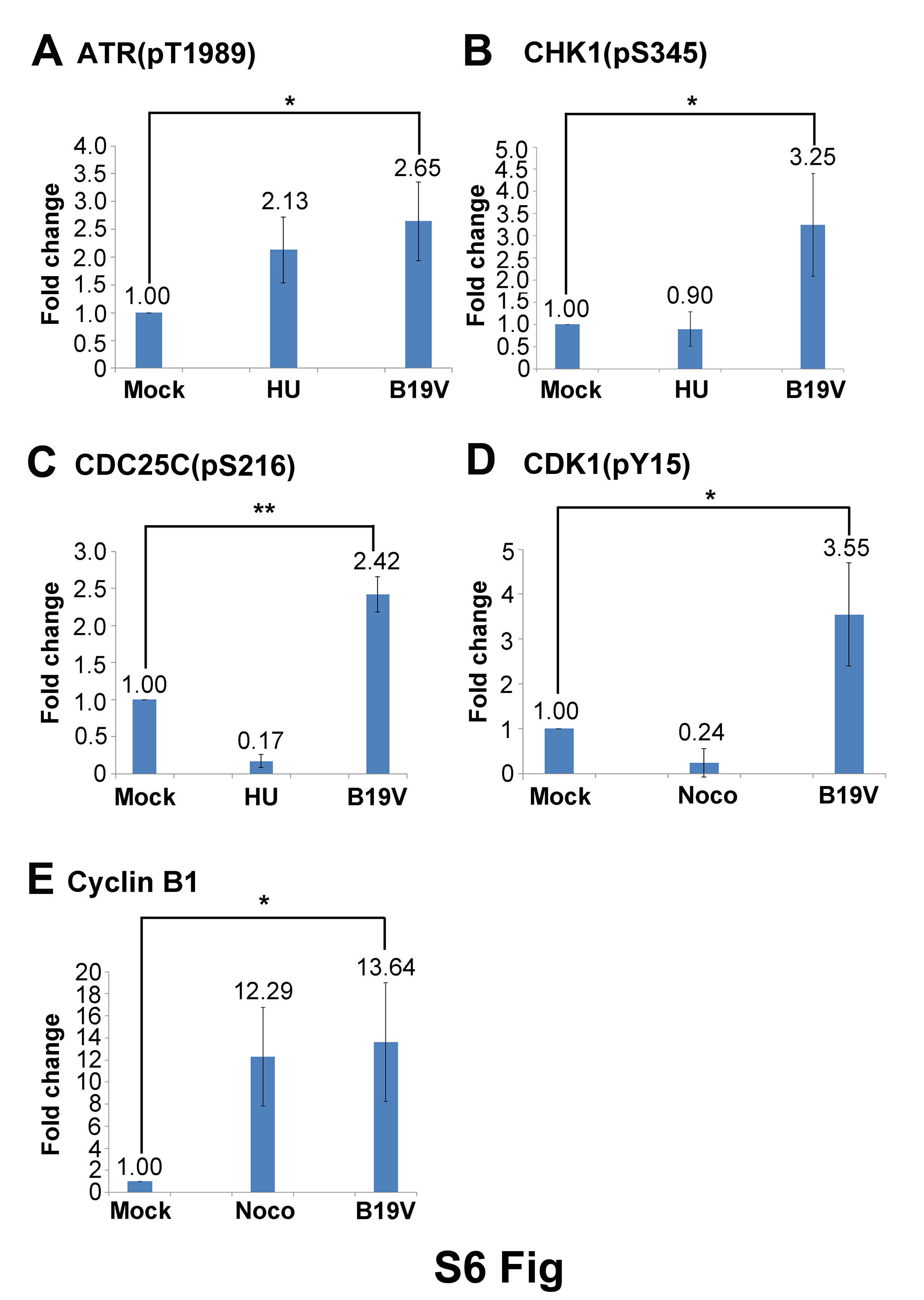

Supplement: S6 Fig — The detected bands on the blots shown in Fig 10A & 10B were quantified as the expression levels of indicated proteins: (A) ATR(pT1989), (B) CHK1(pS345), (C) CDC25C(pS216), (D) CDK1(pY15), and (E) Cyclin B1, and the results are expressed as the mean ± standard deviation of at least three independent experiments. Statistical analysis was performed in paired groups as indicated. **P<0.01 and *P<0.05. (TIF) [file ppat.1006266.s006.tif]

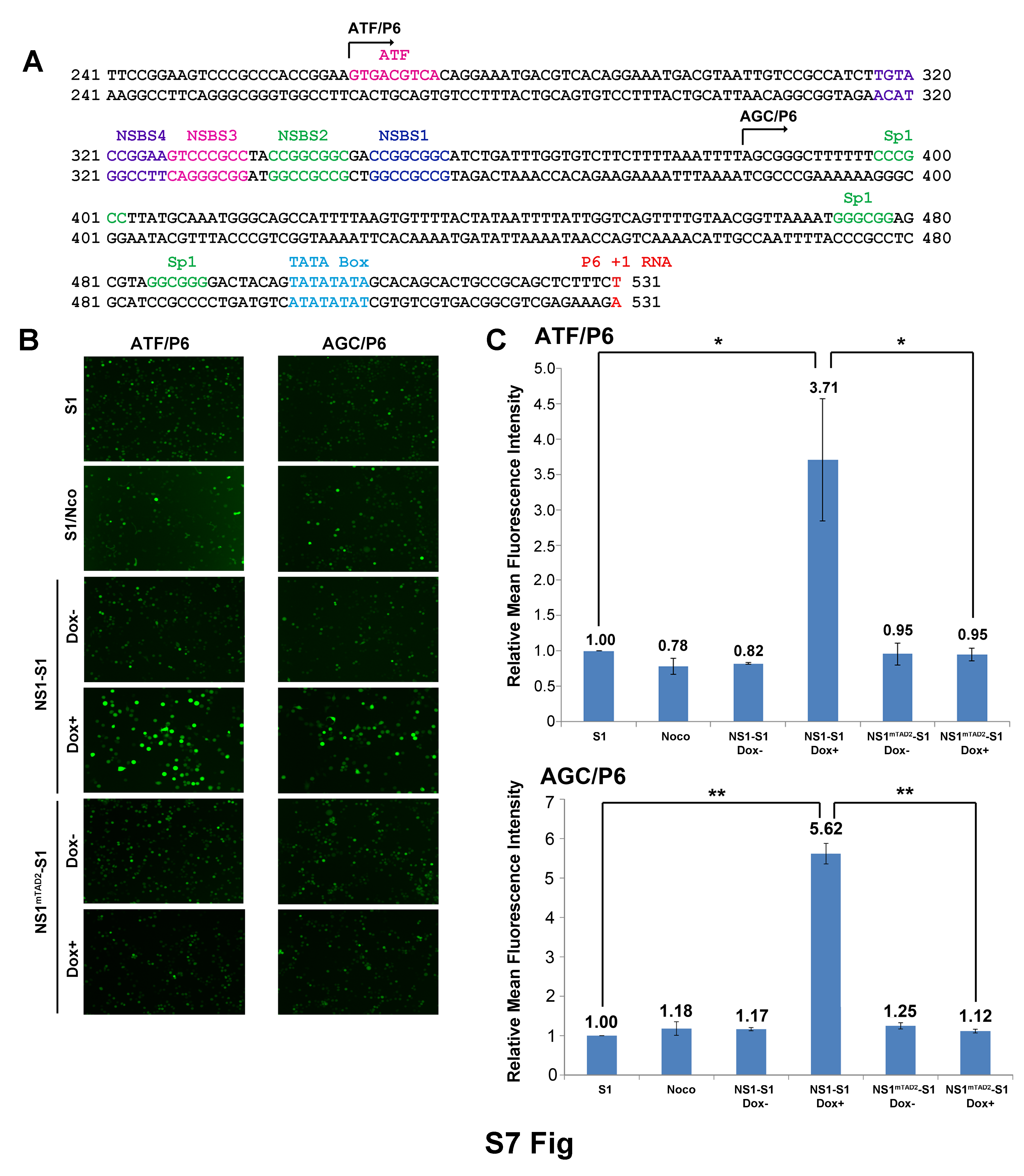

Supplement: S7 Fig — (A) A diagram of the P6 promoter. B19V sequence of nt 241–531 (GenBank accession no.: AY386330) is shown. Important putative motifs on the P6 promoter are highlighted. (B&C) NS1, but not NS1mTAD2, transactivates the P6 promoter. UT7/Epo-S1 (S1) cells, S1 cells treated with nocodazole (S1/Noco), and NS1-S1 and NS1mTAD2-S1 cell lines treated with/without Dox (Dox-/Dox+) were transduced with Lenti-ATF/p6-GFP or Lenti-AGC/p6-GFP. (B) Immunofluorescence analysis. At 48 h post-transduction, cells were observed under a Nikon inverted fluorescent microscope and images were acquired at 10 × magnification. (C) Flow cytometry analysis. At 48 h post-transduction. cells were collected for flow cytometry analysis to determine the mean florescence intensity. Relative mean florescence intensity is shown as the mean ± standard deviation of at least three independent experiments. Paired groups were statistically analyzed. **P<0.01 and *P<0.05. (TIF) [file ppat.1006266.s007.tif]

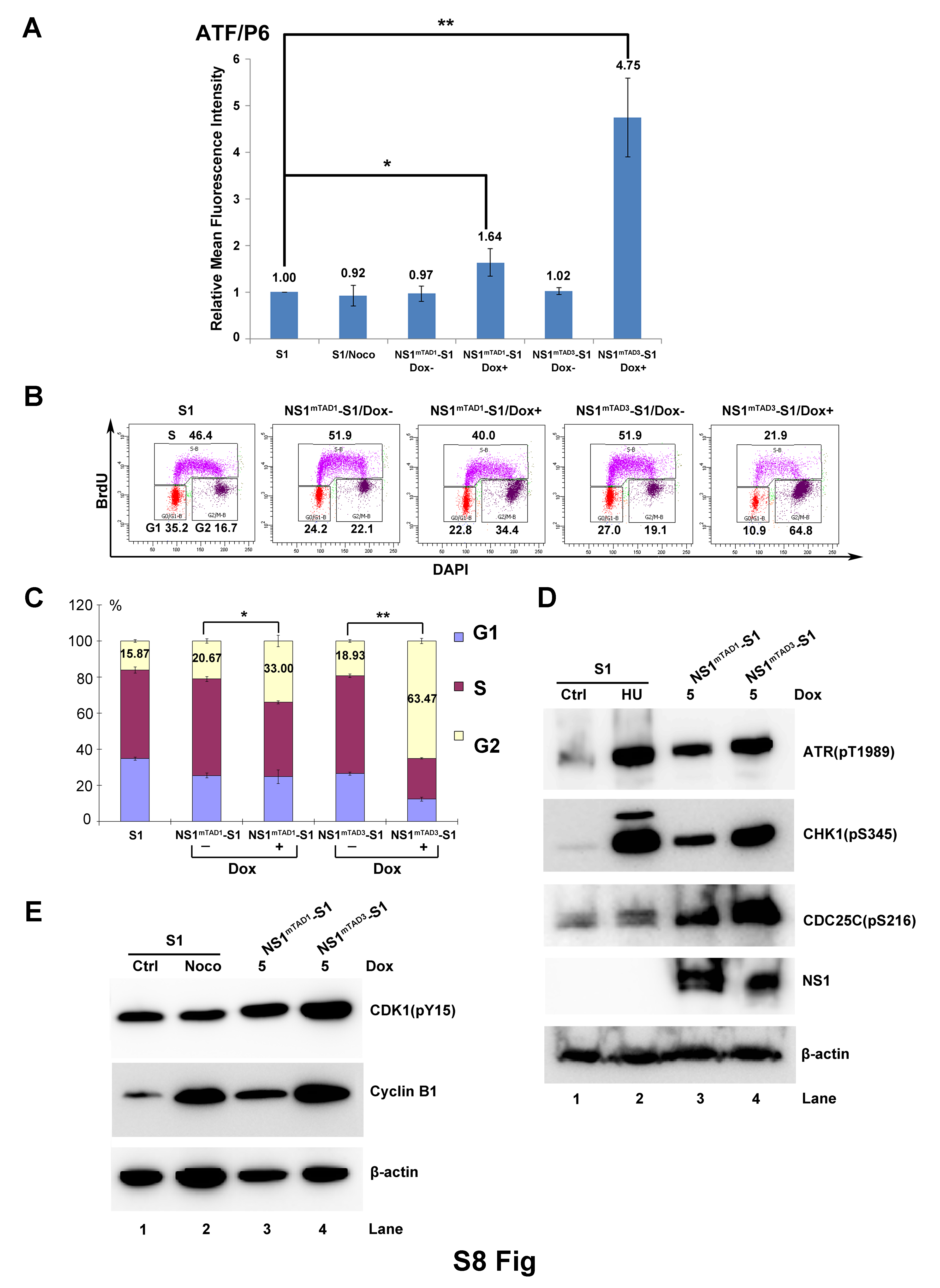

Supplement: S8 Fig — (A) B19V NS1 mutants, NS1mTAD1 and NS1mTAD3, transactivate P6 promoter. UT7/Epo-S1 (S1) cells, S1 cells treated with nocodazole (S1/Noco), and NS1mTAD1-S1 and NS1mTAD3-S1 cell lines treated with/without Dox (Dox-/Dox+) were transduced with Lenti-ATF/p6-GFP. At 48 h post-transduction, cells were collected for flow cytometry analysis to determine the mean florescence intensity. Relative mean florescence intensity is shown as the mean ± standard deviation of at least three independent experiments. Paired groups were statistically analyzed. **P<0.01 and *P<0.05. (B&C) Cell cycle analysis. (B) NS1mTAD1-S1 and NS1mTAD3-S1 cells were treated with Dox (Dox+) or not (Dox-) for 72 h, and then were analyzed for cell cycle using flow cytometry. The numbers shown in each histogram are percentages of the cell populations at G1-, S-, and G2-phase, respectively. (C) Statistical analysis. The percentage of cells at G1, S, and G2 are depicted in color. The percentages of the cells at G2 are shown in numbers, and compared in pairs as shown. *P<0.05, **P<0.01. (D&E) Analysis of the ATR-CHK1-CDC25C-CDK1 pathway. NS1mTAD1-S1 and NS1mTAD3-S1 cells were treated with Dox and collected for lysis 72 h later. (D) Cell lysates were analyzed for expression of phosphorylated ATR, ATR(pT1989), phosphorylated CHK1 and CDC25C, CHK1(pS345) and CDC25C(pS216), and NS1 (using an anti-Strep antibody) by Western blotting. (E) Cell lysates were further analyzed for expression of CDK(pY15) and cyclin B1 by Western blotting. β-actin was used as a loading control. Untreated S1 cells, S1 cells treated with nocodazole (Noco), and S1 cells treated with HU were used as controls. (TIF) [file ppat.1006266.s008.tif]
