# Supplementary material for: Parvovirus B19 NS1 protein induces cell cycle arrest at G2-phase by activating the ATR-CDC25C-CDK1 pathway
Source: PLoS Pathog. 2017 Mar 6;13(3):e1006266. doi: 10.1371/journal.ppat.1006266 (PMC5354443; doi:10.1371/journal.ppat.1006266)
Supplement: S1 Table — Nucleotide sequences used to generate MYT1, MAPKAPK2 (MK2), p38MAPK (p38), p21, MARK3, and ATR lentiviral shRNA vectors are shown from 5’ to 3’ end. Validated shRNA sequences were obtained from Sigma (St. Louis, MO). (DOCX) [file ppat.1006266.s009.docx]

**S1 Table. Sequences of various shRNAs used in the study**

shMYT1-1: 5’-CCG GTC GGT CAC ATC AGC GGG AAA TCT CGA GAT TTC CCG CTG ATG TGA CCG ATT TTT G-3’

shMYT1: 5’-CCG GGC CGA TGA AGA AAG TGC GTT TCT CGA GAA ACG CAC TTT CTT CAT CGG CTT TTT G-3’

shMK2-1: 5’-CCG GCC AGC ACT CGA TTG TTG TAA ACT CGA GTT TAC AAC AAT CGA GTG CTG GTT TTT G-3’

shMK2: 5’-CCG GAG AAA GAG AAG CAT CCG AAA TCT CGA GAT TTC GGA TGC TTC TCT TTC TTT TTT G-3’

shp38-1: 5’-CCG GCC ATG TTC AGT TCC TTA TCT ACT CGA GTA GAT AAG GAA CTG AAC ATG GTT TTT G-3’

shp38: 5’-CCG GCC ATG AGG CAA GAA ACT ATA TCT CGA GAT ATA GTT TCT TGC CTC ATG GTT TTT G-3’;

shp21: 5’-CCG GGA CAG ATT TCT ACC ACT CCA ACT CGA GTT GGA GTG GTA GAA ATC TGT CTT TTT G-3’

shp21-2: 5’-CCG GCG CTC TAC ATC TTC TGC CTT ACT CGA GTA AGG CAG AAG ATG TAG AGC GTT TTT G-3’

shMark3-1: 5’-CCG GGT GGA ATG ACA CGA CGA AAT ACT CGA GTA TTT CGT CGT GTC ATT CCA CTT TTT G-3’

shMark3: 5’-CCG GGG TGA AGT ATT TGA CTA TTT GCT CGA GCA AAT AGT CAA ATA CTT CAC CTT TTT G-3’

shATR: 5’-CCG GGG CGT CGT CTC AGC TCG TCT CCT CGA GGA GAC GAG CTG AGA CGA CGC CTT TTT G-3’
